# The Effectiveness and Influence of COVID-19 Vaccination on Perinatal Individuals and Their Newborns: An Updated Meta-Analysis

**DOI:** 10.1155/cjid/6115890

**Published:** 2025-06-24

**Authors:** Zi-Jin Lei, Min-Xi Bai, Min-Jue Li, Peng Jin, Yu-Bin Ding

**Affiliations:** ^1^Chongqing Medical University, Chongqing 86, China; ^2^School of Statistics, East China Normal University, Shanghai 86, China; ^3^Department of Obstetrics and Gynecology, Women and Children's Hospital of Chongqing Medical University, Chongqing 86, China; ^4^Joint International Research Laboratory of Reproduction and Development of the Ministry of Education of China, School of Public Health, Chongqing Medical University, Chongqing 86, China

**Keywords:** COVID-19 vaccination, neonatal health, obstetric outcomes, pregnancy, vaccine efficacy, vaccine safety

## Abstract

**Background:** The COVID-19 pandemic has disproportionately affected pregnant individuals, increasing risks of severe illness and adverse outcomes. While vaccination is a key mitigation strategy, initial exclusion from clinical trials led to limited safety data. Despite evidence of vaccine effectiveness, hesitancy persists in this population.

**Objective and Sources:** This meta-analysis aims to evaluate the efficacy and impact of COVID-19 vaccination in pregnant individuals, synthesizing evidence from 82 studies (3,676,654 participants) retrieved from PubMed, Embase, Cochrane Library, and Scopus (2019–2024). Study quality was assessed using the Newcastle–Ottawa scale (80/82 scored ≥ 7).

**Key Findings:** Vaccination reduced maternal SARS-CoV-2 infection risk by 48% (odds ratio [OR] = 0.52), with mRNA vaccines showing higher efficacy (52% vs. 43% for inactivated). Maternal hospitalization risk decreased by 42% (OR = 0.58), and severe outcomes by 50% (OR = 0.50). Furthermore, neonatal outcomes improved, including reduced infection (OR = 0.69), preterm birth (OR = 0.87), stillbirth (OR = 0.64), and neonatal death (OR = 0.47). Protection against neonatal death was stronger in individuals without prior infection (OR = 0.43). Third-trimester vaccination may offer better protection against preterm birth.

**Conclusion:** Overall, COVID-19 vaccination during pregnancy effectively mitigates infection and adverse maternal/neonatal outcomes, supporting its clinical recommendation.

## 1. Introduction

The emergence of the COVID-19 pandemic, caused by the novel coronavirus SARS-CoV-2, has presented unprecedented challenges to global public health (https://coronavirus.jhu.edu/map.html). Pregnant individuals, as one of the most vulnerable populations, face increased risks of severe illness and adverse pregnancy outcomes due to COVID-19 [1–6].

Vaccination has been recognized as a critical tool in controlling the spread of COVID-19 and reducing the severity of the disease [7–9] (https://www.gov.uk/government/publications/prioritising-the-first-covid-19-vaccine-dose-jcvi-statement/optimising-the-covid-19-vaccination-programme-for-maximum-short-term-impact) Although the inclusion of pregnant individuals in first clinical trials has historically been limited, resulting in a relative lack of initial data on the effects of COVID-19 vaccines in this population, regulatory agencies and health organizations, including the World Health Organization (WHO) and the Centers for Disease Control and Prevention (CDC), have recommended the vaccination of pregnant individuals based on the benefits observed in the general population and preliminary safety data (https://covid19.who.int/) (https://www.cdc.gov/coronavirus/2019-ncov/need-extra-precautions/pregnant-people.html) (https://www.who.int/director-general/speeches/detail/who-director-general-s-opening-remarks-at-the-media-briefing-on-covid-19---11-march-2020) (https://www.cdc.gov/respiratory-viruses/risk-factors/pregnant-people.html).

Many previous studies have been conducted to demonstrate the effectiveness of COVID-19 vaccines [7, 10–13]. However, skepticism and reluctance remain among a significant portion of pregnant individuals [14–18]. Therefore, a comprehensive study that combines evidence from a longer pandemic period and a larger population is essential, alongside proper guidance and science communication efforts.

The objective of this meta-analysis is to systematically evaluate the existing evidence on the impact and efficacy of COVID-19 vaccination in pregnant individuals. By synthesizing data from January 1, 2019, to October 20, 2024, and stratifying data into multiple subgroups, this analysis aims to provide both a comprehensive overview and a precise interpretation of each outcome associated with COVID-19 vaccination during pregnancy. This includes assessing the incidence of adverse obstetric events and the effectiveness in preventing COVID-19-related morbidity and mortality in both mothers and their infants. Through this meta-analysis, we seek to provide updated, robust evidence to the public. As the benefits of vaccines and vaccine safety continue to influence decision-making among pregnant and lactating individuals, this meta-analysis served as a complement for both professional institutions and individuals. It may help clarify how exactly vaccination affects them, enhance vaccine acceptance, and offer guidance for those who still consider vaccination to protect themselves and their babies, especially as new COVID-19 variants continue to impact people's lives. Additionally, the results may serve as a clue for the protection of pregnant individuals in future pandemic.

## 2. Methods

**Figure 1:**
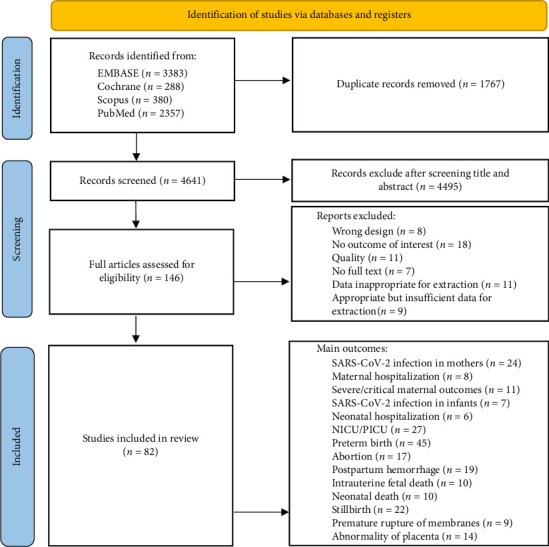
PRISMA flow diagram documenting study identification, screening, and analysis. PRISMA, preferred reporting items for systematic reviews and meta-analyses.

This systematic review and meta‐analysis followed a prospectively registered protocol and was reported in accordance with the PRISMA (Preferred Reporting Items for Systematic Review and Meta‐Analyses) guidelines (Figure 1).

### 2.1. Search Strategy

We systematically searched for studies on pregnant individuals vaccinated against COVID-19 up to October 20, 2024, in the following databases: PubMed, Embase, Cochrane, and Scopus (Table S1). Additionally, the reference lists of relevant studies were reviewed to identify further studies. The language of the included studies was limited to English.

### 2.2. Outcomes and Definitions

Outcomes were categorized into maternal outcomes, neonatal outcomes, and obstetric outcomes. Within each category, outcomes were listed in order of progression:

• Maternal outcomes included maternal infection (confirmed COVID-19 infection), hospitalization, and severe outcomes (such as respiratory failure, septic shock, multiple organ failure, ICU admissions, or death).
• Neonatal outcomes encompassed neonatal infection, hospitalization, neonatal/pediatric intensive care unit (NICU/PICU) admissions, and neonatal death.
• Obstetric outcomes included preterm birth (subdivided into spontaneous preterm birth, iatrogenic preterm birth, and very preterm birth < 34 weeks), preterm premature rupture of membranes (PPROM), placental abnormalities (such as placental abruption, placenta previa, velamentous placenta), and placental pathology (including chorioamnionitis, fetal vascular malperfusion, and maternal vascular malperfusion [MVM]). Other obstetric outcomes included postpartum hemorrhage, abortion, intrauterine fetal death, and stillbirth.
  Additionally, data on obstetric outcomes were stratified by vaccine type (mRNA, inactivated, and adenovirus vaccines), prior infection status, dosing regimen (including booster doses), virus variants, and vaccination timing (first, second, and third trimester) for further analysis.
• Severe/critical maternal outcomes were defined as respiratory failure, septic shock, multiple organ failure, maternal ICU admissions, or death.
  “Mothers with No Prior Infection” was defined as those without documented or confirmed COVID-19 infection during the corresponding period of observation and follow-up.

### 2.3. Data Extraction

A structured data extraction form was used, and data were extracted by multiple reviewers. A minimum of three researchers independently extracted the data and assessed study quality. The data extraction sheet of the search was shared, and all data extracted were cross-checked for discrepancies or missing information and sent for final check by two reviewers. The following study design characteristics were extracted: the first author's name, study setting, time of publication, country of origin, study period, and study design. The documented characteristics of the patients were the total number of patients included, patients included with confirmed COVID-19 vaccination, vaccination types, age of patients in years, and outcomes including maternal infection, maternal hospitalization, critical maternal outcomes (respiratory failure, septic shock, multiple organ failure maternal ICU admissions, or death), neonatal infection, hospitalization, NICU/PICU admissions, preterm birth (< 37 weeks' gestation) and very preterm birth (< 34 weeks' gestation), PPROM, placental morphology abnormalities, placental size abnormalities, placental position abnormalities, placental pathology (chorioamnionitis, fetal vascular malperfusion, and MVM), postpartum hemorrhage, abortion, intrauterine fetal death, stillbirth, and neonatal death.

### 2.4. Study Quality Assessment

The quality of included cohort and case‐control studies was assessed using the Newcastle–Ottawa Scale (NOS), with scores of 0–3, 4–6, and 7–9 considered low, moderate, and high quality, respectively [1]. We mainly included studies with scores of 7–9, which were considered high quality, and only two studies scored 6 as an exception. More details can be found in the Supporting Information (Tables S1 and S2).

### 2.5. Data Synthesis

As we anticipated considerable between-study heterogeneity, we conducted a random-effects meta-analysis using R statistical computing software (v.4.3.3). All analyses were performed using the DerSimonianLaird estimator, which is widely used in meta-analysis for estimating between-study heterogeneity. This method was chosen due to its general applicability and simplicity, particularly in scenarios with a moderate number of studies and when assuming a random effects model. Pooled odds ratios (ORs) and 95% confidence intervals (CIs) were generated for dichotomous outcomes.

The degree of between-study heterogeneity that could not be ascribed to sampling error was evaluated using Chi‐square test and quantified by the *I*^2^ statistic, and was interpreted as low (*I*^2^: < 25%), low to moderate (*I*^2^: 25%–50%), moderate to substantial (*I*^2^: 50%–75%), or substantial (*I*^2^: > 75%). Potential publication bias was assessed using Egger's test and funnel plots for visual inspection when a sufficient number of studies (*N* > 10) were available. Prespecified subgroup analyses were conducted based on prior infection status, type of vaccine, dosing regimen, timing of vaccination, and other factors for results with significant heterogeneity (*I*^2^ > 50%). To assess the leverage of individual studies on the overall estimates, sensitivity analyses were also performed by removing one study at a time. Statistical significance was set at *p* < 0.05 based on two‐sided tests.

## 3. Results

### 3.1. Search Result and Characteristics of Included Studies

Out of the 4641 abstracts screened, 146 studies were selected for full-text review. A total of 82 studies were included, consisting of 72 cohort studies [19–40], [41–60], [61–75], [76–90] and 10 case–control studies [36, 91–99], with 4 studies [36, 60, 95, 98] employing both case–control and cohort designs. These studies involved a total of 3,676,654 participants. In the cohort studies, all control groups consisted of unvaccinated pregnant individuals, while in the case–control studies, data for unvaccinated individuals were extracted from different group classifications. The studies were published between 2021 and 2024, and data were collected from a wide range of countries, including the USA, Canada, UK, China, Israel, South Korea, Thailand, Singapore, India, Iran, Turkey, Scotland, Sweden, Norway, Germany, Australia, Mexico, and Qatar. The included vaccines were mRNA, viral vector, and inactivated virus vaccines, which provide a comprehensive perspective on their overall impact. Further details on vaccine types and dosing regimens are summarized in the characteristics table (Table 1).

### 3.2. Meta-Analysis Results

#### 3.2.1. Maternal Outcomes

**Figure 2:**
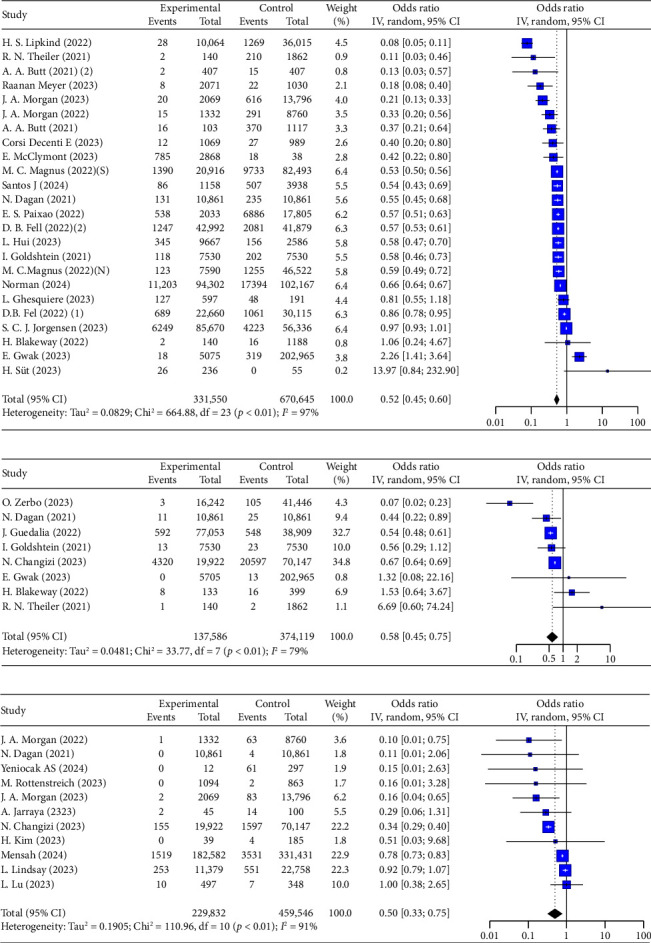
Forest plots showing the results of meta-analyses for maternal outcomes. (a) Infections in mother, (b) maternal hospitalization, (c) severe maternal outcomes.

Overall pooled analyses showed a 50% reduction in the risk of SARS-CoV-2 infection among pregnant individuals (OR = 0.52, 95% CI: 0.45–0.60, *I*^2^ = 97%, *p* < 0.01; 24 studies, 1,002,195 pregnant individuals, Figure 2(a)). Differences in geographical regions, vaccine types, and dosing regimens may partly explain the high heterogeneity. Both mRNA vaccines (OR = 0.48, 95% CI: 0.34–0.67, *I*^2^ = 95%, Table 2) and inactivated vaccines (OR = 0.57, 95% CI: 0.51–0.63, *I*^2^ = 0%, Table 2) were effective in reducing the risk of COVID-19 infection among pregnant individuals. In the dosing regimen subgroup, a greater reduction in risk was observed with two doses (OR = 0.40, 95% CI: 0.24–0.65, *I*^2^ = 95%) compared to one dose (OR = 0.77, 95% CI: 0.67–0.88, *I*^2^ = 0%, Table 2). A booster dose provided an additional 44% protection compared to the original vaccine series (booster vs. two doses: OR = 0.56, 95% CI: 0.48–0.66, Figure S1).

Collectively examined 137,586 experimental events (vaccinated pregnant individuals) and 374,119 control events (nonvaccinated pregnant individuals), a significant association was found between maternal hospitalization and vaccination in the overall analysis (OR = 0.58, 95% CI: 0.45–0.75, *I*^2^ = 79%, *p* < 0.01; 8 studies, 511,705 pregnant individuals, Figure 2(b)). The inactivated vaccines showed a stronger effect (OR = 0.20, 95% CI: 0.06–0.60, *I*^2^ = 81%, Table 2). A booster dose provided further protection (booster vs. unvaccinated: OR = 0.42, 95% CI: 0.36–0.49, Figure S3). In five studies, hospitalization was primarily attributed to COVID-19 infection [24, 40, 41, 55, 96], while in the other three studies [38, 42, 89], the direct reason was not clearly defined.

However, the inactivated vaccine group (OR = 11.19, 95% CI: 10.53–11.89, *I*^2^ = 0%, Table 2, Sinopharm Biotech) and adenovirus vaccine group (OR = 10.97, 95% CI: 8.68–13.87, *I*^2^ = 0%, Table 2, COVISHIELD and Sputnik V [rAd26 and rAd5]) showed a statistically significant increased risk of hospitalization compared to unvaccinated pregnant individuals.

We also looked into the vaccine effectiveness against different variants. Protection was more stable during the Omicron waves (Delta wave vs. Omicron wave: OR = 0.75, 95% CI: 0.64–0.89, Figure S4 vs. OR = 0.10, 95% CI: 0.01–0.73, Figure S5). For both variants, a booster dose showed a positive outcome (Figures S6 and S7).

There was a significant reduction in severe/critical maternal outcomes (defined as respiratory failure, septic shock, multiple organ failure, maternal ICU admissions, or death) (OR = 0.50, 95% CI: 0.33–0.75, *I*^2^ = 91%, Figure 2(c)). However, no significant differences were observed between different vaccine types (Table 2).

More detailed results can be found in Table 2 and Figures S1–S8.

#### 3.2.2. Neonatal Outcomes

**Figure 3:**
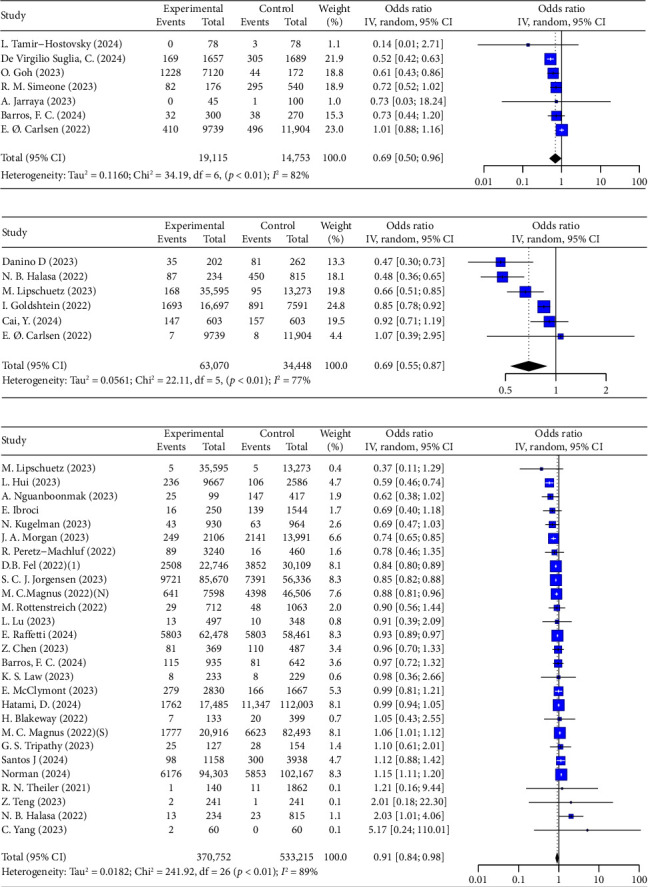
Forest plots showing the results of meta-analyses for neonatal outcomes. (a) Infant infection, (b) neonatal hospitalization, (c) NICU (neonatal intensive care unit).

Vaccination was associated with a reduced risk of neonatal or infant infection during delivery hospitalization and early months of life (OR = 0.69, 95% CI: 0.50–0.96, *I*^2^ = 82%, *p* < 0.01; 7 studies, 33,868 cases, Figure 3(a)), with inactivated vaccines showing a similar effect (OR = 0.59, 95% CI: 0.48–0.73, *I*^2^ = 14%, Table 3). Additionally, a 31% reduction in the odds of hospitalization was observed among neonates or infants born to vaccinated mothers compared to those born to unvaccinated mothers (OR = 0.69, 95% CI: 0.55–0.87, *I*^2^ = 77%, *p* < 0.01; 6 studies, 97,518 cases, Table 3, Figure 3(b)). Most studies clearly identified hospitalization due to COVID-19, except for two studies [82, 94].

A 9% reduction was also observed in the NICU/PICU admissions during delivery hospitalization (OR = 0.91, 95% CI: 0.84–0.98, *I*^2^ = 89%, *p* < 0.01; 27 studies, 903,967 cases, Table 3, Figure 3(c)).

In the NICU/PICU subgroup analysis, no differences were detected between different vaccination times during the first, second, or third trimester (Table 3). mRNA vaccines showed a protective effect (OR = 0.82, 95% CI: 0.70–0.98), whereas inactivated vaccines demonstrated no significant effect (OR = 0.99, 95% CI: 0.94–1.05, *I*^2^ = 95%). A complete series of two doses was found to be effective (OR = 0.91, 95% CI: 0.84–0.99, *I*^2^ = 78%), while one dose showed no protective benefit (OR = 1.04, 95% CI: 0.94–1.14, *I*^2^ = 75%). Two studies included data from the Delta and Omicron periods, and no significant relationship was found between vaccine timing and neonatal outcomes during these variants.

Further details can be found in Table 3 and Figures S9–S14.

#### 3.2.3. Obstetric Outcomes

**Figure 4:**
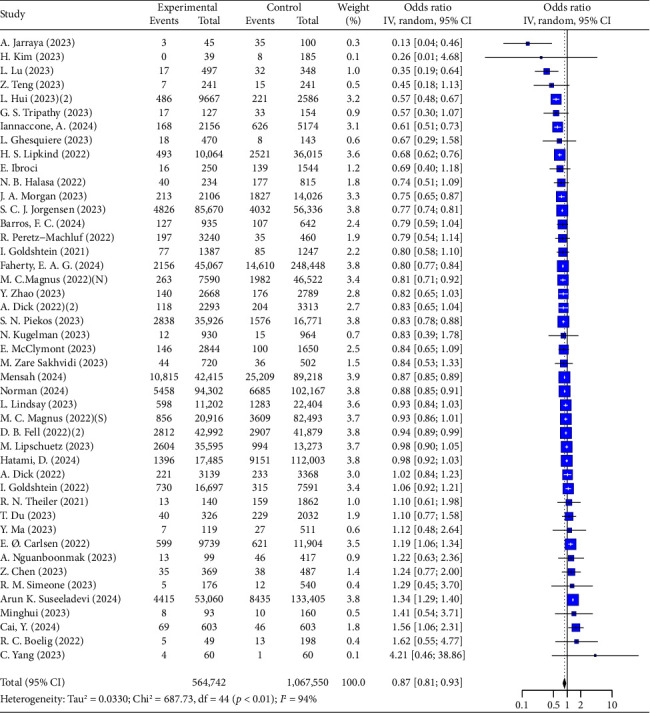
Forest plots showing the results of meta-analyses for preterm birth.

The analysis of preterm birth outcomes associated with COVID-19 vaccination during pregnancy revealed a consistent protective effect. Overall, vaccination reduced the risk of preterm birth by 13% (OR = 0.87, 95% CI: 0.81–0.93, *I*^2^ = 94%, Table 4, Figure 4). This effect was more pronounced in individuals with prior infection (OR = 0.82, 95% CI: 0.74–0.90, *I*^2^ = 96%, Table 4) compared to those without prior infection (OR = 0.93, 95% CI: 0.81–0.93, *I*^2^ = 66%). However, the impact of inactivated and mRNA vaccines was inconclusive (Inactivated: OR = 0.95, 95% CI: 0.79–1.16, *I*^2^ = 61%; mRNA: OR = 0.99, 95% CI: 0.70–1.40, *I*^2^ = 99%, Table 4), while the adenovirus vaccine was associated with an increased risk (OR = 1.53, 95% CI: 1.14–2.06, *I*^2^ = 99%, Table 4). Two doses of the vaccine significantly reduced the risk of preterm birth (OR = 0.80, 95% CI: 0.71–0.90, *I*^2^ = 84%), but a booster dose did not show additional benefit (OR = 0.99, 95% CI: 0.44–2.22, Figure S16). The subgroup analysis of vaccination timing recommended vaccination in the third trimester (OR = 0.76, 95% CI: 0.66–0.88, *I*^2^ = 99%, Table 4). Notably, vaccination was particularly effective in preventing very preterm births (OR = 0.70, 95% CI: 0.57–0.86) and spontaneous preterm births (OR = 0.76, 95% CI: 0.67–0.86, *I*^2^ = 85%). More results are available in Table 4 and Figures S16 and S17.

**Figure 5:**
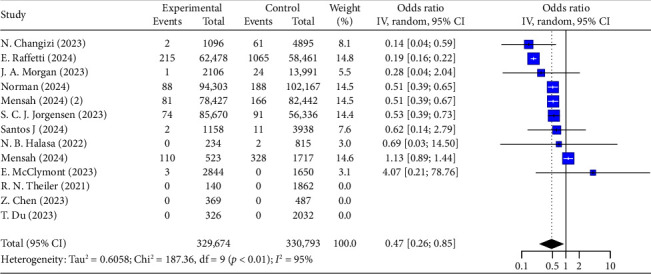
Forest plots showing the results of meta-analyses for neonatal death.

Concerning the risk of neonatal death, the analysis shows that COVID-19 vaccination during pregnancy is associated with a 53% reduction (OR = 0.47, 95% CI: 0.26–0.85, *I*^2^ = 95%, Figure 5). This protective effect is evident in individuals without prior infection (OR = 0.44, 95% CI: 0.22–0.86, *I*^2^ = 97%, Table 5), but is less significant in those with prior infection (OR = 0.56, 95% CI: 0.28–1.09, *I*^2^ = 81%, Table 5).

**Figure 6:**
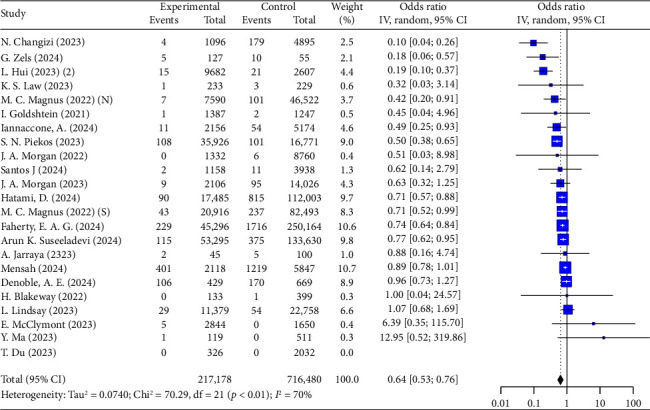
Forest plots showing the results of meta-analyses for stillbirth.

Furthermore, the results indicate that COVID-19 vaccination during pregnancy significantly reduces the risk of stillbirth (OR = 0.64, 95% CI: 0.53–0.76, *I*^2^ = 70%, Figure 6), with a more pronounced protective effect in individuals with prior infection (OR = 0.62, 95% CI: 0.50–0.76, Table 5).

Other analyses revealed no significant effect on the risk of PPROM, placental abnormalities, intrauterine fetal death, or abortion. Inactivated vaccines appear to be likely effective in reducing postpartum hemorrhage (OR = 0.49, 95% CI: 0.24–1.00, *I*^2^ = 57%, Table 5).

Detailed results, including overall analyses and subgroup outcomes, can be found in Table 5 and Figures S15 and S18–S21.

### 3.3. Publication Bias

Potential publication bias was assessed using Egger's test and the creation of funnel plots for visual inspection, when a sufficient number of studies (*N* > 10) were available. No significant evidence of publication bias was found, except for studies on placental abnormalities, where two statistical outliers were excluded to address the bias.

## 4. Discussion

Our systematic review and meta-analysis, which included 3,676,654 individuals, primarily assessed the effectiveness of COVID-19 vaccinations and their impact on pregnancy outcomes. COVID-19 vaccination was associated with a reduced risk of maternal COVID-19 infection (OR = 0.52, 95% CI: 0.45–0.60, *I*^2^ = 97%), maternal hospitalization (OR = 0.58, 95% CI: 0.45–0.75, *I*^2^ = 79%), severe maternal outcomes (OR = 0.50, 95% CI: 0.33–0.75), and adverse neonatal outcomes, particularly neonatal death (OR = 0.47, 95% CI: 0.26–0.85, *I*^2^ = 91%), stillbirth (OR = 0.64, 95% CI: 0.53–0.76, *I*^2^ = 70%), neonatal infection (OR = 0.69, 95% CI: 0.50–0.96, *I*^2^ = 82%), neonatal hospitalization (OR = 0.69, 95% CI: 0.55–0.87, *I*^2^ = 77%), preterm birth (OR = 0.87, 95% CI: 0.81–0.93, *I*^2^ = 94%), and NICU/PICU admission (OR = 0.91, 95% CI: 0.84–0.98, *I*^2^ = 89%).

No significant increase or decrease in the risk of other adverse outcomes was found.

Overall, no adverse impacts were observed.

The third trimester appeared to be an optimal time for vaccination to reduce the risk of preterm birth, while vaccination timing had no significant effect on other outcomes.

No evidence of considerable publication bias was tested found, except for studies on placental abnormalities, which were addressed by excluding two statistical outliers.

### 4.1. Preterm Birth

The significant reduction in maternal COVID-19 infection rates highlights the vaccine's role in preventing severe illness during pregnancy, which is crucial given the increased risk of complications for pregnant individuals.

Previous studies have reported that COVID-19 infection during pregnancy significantly elevates the risk of preterm birth [4, 100, 101] and other neonatal complications [102]. This risk is often influenced by the severity of the infection, with more severe cases leading to higher rate [103, 104]. However, other studies have found no significant change, or even a decrease, in preterm birth rates during the pandemic [105, 106].

While the effects of COVID-19 on pregnancy are well documented, investigating how COVID-19 vaccination impacts this population remains important, particularly in light of ongoing concerns that contribute to vaccine hesitancy, which may pose risks to both individuals and their babies. The observed 13% reduction in preterm labor rates could suggest a potential link between SARS-CoV-2 infection and preterm birth. To further explore the underlying mechanisms, our study also examined the risk of PROM and placental abnormalities, as several studies have reported pathological changes, placental infections, and insufficiency following COVID-19 infection in pregnant individuals [107–110]. However, no significant reduction in risk was found among vaccinated individuals (PROM: OR = 0.94, 95% CI: 0.69–1.29; abnormality of placenta: OR = 0.86, 95% CI: 0.64–1.17, Table 5). While a possible adverse effect of chorioamnionitis was observed, the result was not significant (OR = 1.35, 95% CI: 0.94–1.92). This suggests that the exact mechanisms by which COVID-19 leads to adverse outcomes remain unclear. Our analysis included different types of preterm birth, with consistent protective effects observed for both spontaneous and initiated preterm birth (Table 4).

### 4.2. Neonatal Death and Stillbirth

The reduction in neonatal death and stillbirth rates supports the notion that vaccination lactating individuals may provide additional protection to the fetus through the transfer of maternal antibody, including transplacental transfer and breastfeeding. Studies have shown increased levels of Spike IgG and SIgA antibodies in breast milk, while maternal IgG antibodies to SARS-CoV-2 are efficiently transported across the placenta [111, 112] This protection appears to remain relatively significant regardless of the timing of vaccination.

### 4.3. Infection Status

The variation in vaccine effectiveness based on infection status underscores the complexity of immune responses during pregnancy, suggesting that both natural immunity and vaccine-induced immunity [113, 114] contribute to improved outcomes. The impact of COVID-19 further complicates the situation. To sorely looking into the independent effect of vaccination, subgroup 1 is established. Most results from the vaccine-induced group continue to support the protective effect against adverse perinatal outcomes. The protective effect of vaccination remains constant despite the potential risks COVID-19 may pose to pregnancies, even after excluding the influence of natural immunity in pregnant individuals with unclear prior infection status. However, instability was observed in outcomes like preterm birth and neonatal death, where vaccination efficacy was more pronounced in those without prior infection during pregnancy. This suggests that COVID-19 infection may contribute more to adverse outcomes than natural immunity.

### 4.4. Inactivated Vaccines and Adenovirus Vaccines

Inactivated vaccination effectively reduces the rate of maternal infection by 48%, maternal hospitalization by 42%, and neonatal infection by 31%. It also marginally lowered the odds of postpartum hemorrhage. However, for other outcomes, no consistent improvement was observed compared to the unvaccinated control group.

Data on adenovirus vaccines were collected for outcomes related to preterm birth, abortion, and stillbirth. Analysis of other outcomes was not conducted due to the limited number of studies or lack of specific data on this vaccine type, as most studies involved a mixed-vaccination population. No significant impact was observed, except for preterm birth, where the OR for the adenovirus vaccine was 1.53 (95% CI: 1.14–2.06).

Overall, the findings support the continued use of inactivated vaccines to protect maternal and neonatal health during the ongoing pandemic, despite some variations in the protective effects on different maternal and neonatal outcomes with different vaccine types.

### 4.5. Booster Dose

Booster doses can increase both sIgA and IgG antibody titers, enhancing immunization in neonates of breastfeeding mothers and providing additional protection through increased immunoglobulin transfer via the cord blood [115–117]. Studies have shown that licensed mRNA COVID-19 vaccines maintain robust immunity for approximately 5 to 6 months [116], making booster doses necessary to sustain SARS-CoV-2-specific responses and address the emergence of more contagious variants [118, 119] (https://www.cdc.gov/media/releases/2021/s1129-booster-recommendations.html). Several studies have demonstrated the safety of booster doses during pregnancy.

For further investigation, this subgroup was analyzed across various outcomes, including maternal COVID-19 infection, maternal hospitalization, preterm birth, PROM or placental abruption, abnormalities of the placenta, postpartum hemorrhage, abortion, intrauterine fetal death, neonatal death, and stillbirth. The results show either no significant effect or weak promotion of outcomes, which suggest that while full vaccination provided more robust protection, the benefit of a booster dose was less clear. The absence of significant adverse effects indicates that booster doses are likely safe. However, the high variability in certain outcomes (e.g., maternal hospitalization and postpartum hemorrhage) highlights the need for more robust studies with larger sample sizes to confirm these findings.

### 4.6. Comparison With Existing Literature

A 2024 meta-analysis [120] reported a substantial reduction in maternal hospitalization (OR = 0.06, 95% CI: 0.01–0.71) in its primary analysis of test-negative design studies. However, in the comparative cohort (unadjusted) studies, the association was inconsistent (OR = 0.41, 95% CI: 0.13–1.28). Based on the adjusted estimates from the extracted data, no association was found between COVID-19 vaccination and other maternal outcomes, except for a reduction in the risk of hypertensive disorders during pregnancy.

A meta-analysis [121] (30 studies involving 862,272 individuals) reported a 1.78-fold increased risk of neonatal infection in the first 2, 4, and 6 months of life during the Omicron period. However, a decrease in maternal hospitalization, ICU admissions, NICU admissions, stillbirth, and preterm births at 37, 32, and 28 weeks' gestation was observed. Our study offers a further and more detailed analysis of potential influencing factors based on a larger population and a broader time range. It suggests that the observed reduction in preterm birth rates is likely attributed to vaccination alone, with prior infection not contributing to the improvement in rates.

A recent meta-analysis [122] reviewed on the efficacy of different types of COVID-19 vaccines in the general population. The findings revealed that mRNA vaccines were the most effective (OR = 0.08, 95% CI: 0.06–0.10), followed by inactivated vaccines (OR = 0.20, 95% CI: 0.14–0.29), such as BBV152 (Bharat Biotech) and CoronaVac (Sinovac). Nonreplicating viral vector–based vaccines, including ChAdOx1 nCoV-19 (AstraZeneca/Oxford Covishield), Ad26.COV2.S (Janssen), and Ad5-nCoV (CanSino), demonstrated moderate efficacy (OR = 0.36, 95% CI: 0.28–0.46).

A 2023 meta-analysis [123] looked further into the effects of dose regimen and timing of vaccination during pregnancy, confirming the dose- and time-dependent effectiveness of COVID-19 vaccines in pregnant individuals. Vaccine effectiveness increased from day 11 to day 28 after the first dose, with an overall efficacy of approximately 96% after two doses. For inactivated vaccines, effectiveness was 40.97% after one dose (≥ 14 days) and 85.39% after full vaccination. These findings may help explain why, in our analysis, vaccination during the third trimester significantly reduces the likelihood of preterm birth. As previously observed, maternal infection is a major contributor to adverse perinatal outcomes, including preterm birth. Recent vaccination improves immunity, leading to a reduction in preterm birth rates. Additionally, no positive or adverse effects on perinatal outcomes (such as placental abruption, postpartum hemorrhage, miscarriage, stillbirth, preterm birth, or small-for-gestational-age infants) were found in this study.

### 4.7. Limitations

This analysis has several limitations. The inclusion of studies with varying designs, populations, and definitions of outcomes contributed to heterogeneity in some results. Moreover, although connections between comorbidities—such as preexisting diabetes mellitus, hypertension, and cardiovascular disease—and an increased risk of adverse pregnancy outcomes have been identified [124], we could not adjust for all potential biases due to incomplete information regarding the populations included.

The lack of data for certain subgroups, such as the specific effects of booster doses, limits the generalizability of some findings. Additionally, the observational nature of most included studies prevents us from establishing causality definitively.

### 4.8. Implications for Practice

Despite these limitations, the findings strongly support the recommendation for COVID-19 vaccination during pregnancy to protect both maternal and fetal health. Healthcare providers should actively encourage vaccination among pregnant individuals, emphasizing the safety and potential benefits in preventing severe COVID-19 and improving neonatal health.

### 4.9. Future Research

Future studies should constantly focus on large, diverse populations to validate these findings and explore the mechanisms underlying the protective effects of vaccination. Moreover, further research is needed to assess the effects of adenovirus vaccines and booster doses in pregnant populations. Long-term outcomes of vaccinated pregnancies, as well as the potential benefits of more specific vaccine timing, should also be investigated to provide a more comprehensive understanding of vaccination's impact on maternal and fetal health.

## 5. Conclusion

This meta-analysis confirms that COVID-19 vaccination during pregnancy is safe and beneficial, reducing the risks of maternal infection and several adverse neonatal outcomes. These results support public health policies advocating for the vaccination of pregnant women as a critical measure to protect maternal and fetal health during the pandemic.

## Figures and Tables

**Table 1:** Characteristics of all included study.

Study	Design	Country	Group	Maternal age (year)	Vaccine types	Study period	Outcome measures	NOS
Guedalia et al. (2022)	Cohort	Israel	V: 77,053 UV: 38,909	NA	Pfizer-BioNTech	2021.08.01–2022.03.22	Maternal hospitalization	9

Butt et al. (2021)	Cohort	Qatar	V: 103 UV: 1117	NA	mRNA vaccine	2020.12.20–2021.05.30	Maternal infection	9

Butt et al. (2021) (2)	Test-negative, case–control	Qatar	V: 407 UV: 407	NA	mRNA vaccine	2020.12.20–2021.05.30	Maternal infection	9

Dick et al. (2022)	Retrospective cohort	Israel	V: 3139 UV: 3368	V: 30 ± 4 UV: 30 ± 4	mRNA vaccine	2021.07–2021.10	Postpartum hemorrhage, intrauterine fetal death, preterm birth	9

Dick et al. (2022) (2)	Cohort	Israel	V: 2293 UV: 3313	V: 30 ± 4 UV: 30 ± 4	mRNA vaccine	2020.12–2021.07	Postpartum hemorrhage, intrauterine fetal death, preterm birth	8

Jarraya et al. (2023)	Prospective observational	Tunisia	V: 45 UV: 100	V: 31.64 ± 3.4 UV: 30.61 ± 2.9	Pfizer-BioNTech	2021.01–2022.05	Preterm birth, postpartum hemorrhage, stillbirth, neonatal/infant infection, maternal ICU admission/death, severe or critical maternal COVID-19	8

Nguanboonmak et al. (2023)	Retrospective cohort	Thailand	V: 99 UV: 417	NA	Lacking detailed information	2021.09–2022.04	NICU, postpartum hemorrhage, placental previa, chorioamnionitis, preterm birth	9

Calvert et al. (2022)	Population-based matched cohort	UK	V: 18,780 UV: 18,780	V: 31 ± 18.5 UV: 31 ± 20	Moderna, Pfizer-BioNTech, adenovirus vaccine	2015.01.01–2021.09.28	Spontaneous abortion	9

Yang et al. (2023)	Prospective observational cohort	China	V: 60 UV: 60	V: 31.12 ± 4.47 UV: 28.88 ± 4.29	Sinovac Biotech, Sinopharm Biotech	2021.01.01–2021.12.31	Postpartum hemorrhage, NICU, preterm birth	7

Condon et al. (2023)	Retrospective	USA	V: 502 UV: 12,767	NA	Moderna, Pfizer-BioNTech, adenovirus vaccine	2020.03.28–2022.01.25	Intrauterine fetal death	8

Corsi Decenti et al. (2023)	Prospective cohort	Italy	V: 1069 UV: 989	NA	mRNA vaccine	2022.1.1–2022.5.31	Maternal infection	8

Fell et al. (2022) (2)	Retrospective cohort	Canada	V: 42,992 UV: 41,879	V: 32.9 ± 4.4 UV: 31.3 ± 5.3	Moderna, Pfizer-BioNTech, adenovirus vaccine	2021.05.01–2021.12.31	Maternal infection, preterm birth	8

Fell et al. (2022)	Population-based retrospective cohort	Canada	V: 22,660 UV: 30,115	V: 32.8 ± 4.3 UV: 31.0 ± 5.5	Moderna, Pfizer-BioNTech	2020.12.14–2021.09.30	Maternal infection, NICU, postpartum hemorrhage, chorioamnionitis	8

Danino et al. (2023)	Retrospective, multicenter, case–control, test-negative	Israel	V: 202 UV: 262	NA	Pfizer-BioNTech	2021.3.1–2021.11.31	Neonatal/infant hospitalization	8

Gwak et al. (2023)	Cohort	Korea	V: 5284 UV: 21,136	NA	Moderna, Pfizer-BioNTech, adenovirus vaccine	2021.01.01–2021.12.31	Spontaneous abortion, maternal infection, maternal hospitalization	9

Ibroci et al. (2023)	Prospective cohort	USA	V: 250 UV: 1544	NA	mRNA vaccine, adenovirus vaccine	2020.04–2022.02	Spontaneous abortion, intrauterine fetal death, NICU, preterm birth	8

McClymont et al. (2023)	Prospective (but some was retrospective)	Canada	V: 2868 UV: 1660	NA	Moderna, Pfizer-BioNTech, adenovirus vaccine	2021.07–2022.06.10	Spontaneous abortion, stillbirth, neonatal death, maternal infection, NICU, preterm birth	9

Carlsen et al. (2022)	Register-based cohort	Norway	V: 9739 UV: 11,904	V: 31.2 ± 2.95 UV: 30.2 ± 3.35	mRNA vaccine	2021.09.01–2022.02.28	Neonatal/infant infection, neonatal/infant hospitalization, preterm birth	9

Kharbanda et al. (2021)	Case–control	USA	V: 21,267 UV: 264,104	NA	Moderna, Pfizer-BioNTech, adenovirus vaccine	2020.12.15–2021.06.28	Spontaneous abortion	8

Ozer et al. (2023)	Retrospective cohort	Turkey	V: 18 UV: 20	V: 29.7 ± 3.7 UV: 29.5 ± 5.0	Pfizer-BioNTech	2021.09–2021.11	Placental pathology	7

Paixao et al. (2022)	Test-negative case–control	Brazil	V: 2033 UV: 17,805	NA	Sinovac Biotech	2021.03.15–2021.10.03	Maternal infection	9

Shanes et al. (2021)	Prospective	USA	V: 84 UV: 116	V: 33.7 ± 3.1 UV: 32.5 ± 4.8	Lacking detailed information	2020.04–2021.04	Fetal vascular malperfusion	7

Tripathy et al. (2023)	Retrospective cohort	India	V: 127 UV: 154	V: 28.24 ± 4.1 UV: 28.12 ± 4.6	Covaxin, adenovirus vaccine	2021.10–2021.12.31	Intrauterine fetal death, PPROM premature rupture of membranes/placental abruption, NICU, preterm birth	8

Vazquez-Benitez et al. (2023)	Case–control	USA	V: 15,108 UV: 90,338	NA	Moderna, Pfizer-BioNTech, adenovirus vaccine	2020.12.15–2021.06.28	Spontaneous abortion	8

Blakeway et al. (2022)	Retrospective cohort	UK	V: 133 UV: 399	V: 35 ± 2.6 UV: 33 ± 3	Moderna, Pfizer-BioNTech, adenovirus vaccine	2020.03.01–2021.07.04	Postpartum hemorrhage, stillbirth, PROM (premature rapture of membranes), suspected chorioamnionitis, maternal infection, NICU, maternal hospitalization	8

Kim et al. (2023)	Retrospective cohort	Korea	V: 39 UV: 185	NA	Lacking detailed information	2020.11.01–2022.03.07	Maternal ICU admission/death, preterm birth	6

Lipkind et al. (2022)	Retrospective cohort	USA	V: 10,064 UV: 36,015	V: 32.3 ± 4.5 UV: 29.8 ± 5.3	Moderna, Pfizer-BioNTech, adenovirus vaccine	2020.05.17–2021.07.31	Maternal infection, preterm birth	8

Süt et al. (2023)	Prospective cohort	Turkey	V: 236 UV: 55	V: 29.5 ± 5.0 UV: 27.5 ± 4.7	Sinovac Biotech, Pfizer-BioNTech	2022.01–2022.04	Maternal infection	7

Goldshtein et al. (2021)	Retrospective cohort	Israel	V: 7530 UV: 7530	V: 31.1 ± 5.01 UV: 31.0 ± 4.85	Pfizer-BioNTech	2020.12.19–2021.02.28	Spontaneous abortion, stillbirth, maternal infection, maternal hospitalization, preterm birth	7

Teng et al. (2023)	Retrospective-prospective cohort	China	V: 241 UV: 241	NA	Inactivated vaccine	2021.05–2021.11	Spontaneous abortion, Intrauterine fetal death, PPROM premature rupture of membranes/placental abruption, placental previa, NICU, preterm birth	7

Chen et al. (2023)	Cohort	China	V: 369 UV: 487	V: 31.50 ± 4.26 UV: 31.49 ± 4.27	Sinovac Biotech, Sinopharm Biotech	2022.03.01–2022.06.30	Neonatal death, mixed placenta, NICU, preterm birth	9

Zhao et al. (2023)	Cohort	China	V: 2668 UV: 2789	NA	Inactivated vaccine	Unknown-2022.07.15	Preterm birth	9

Ma et al. (2023)	Prospective cohort	China	V: 165 UV: 717	NA	Sinovac Biotech, Sinopharm Biotech	2021.07.01–2022.05.30	Spontaneous abortion, stillbirth, preterm birth	7

Wainstock et al. (2021)	Retrospective cohort	Israel	V: 913 UV: 3486	V: 30.6 ± 5.3 UV: 28.2 ± 5.7	mRNA vaccine	2021.01–2021.06	Postpartum hemorrhage, PROM	9

Du et al. (2023)	Retrospective cohort	China	V: 389 UV: 2269	V: 30.85 UV: 27.9	Inactivated vaccine	2021.01–2021.10	Postpartum hemorrhage, spontaneous abortion, stillbirth, neonatal death, preterm birth	9

Piekos et al. (2023)	Retrospective cohort	USA	V: 35,926 UV: 16,771	NA	mRNA vaccine	2021.01.26–2022.10.26	Stillbirth, preterm birth	9

Jorgensen et al. (2023)	Population-based retrospective cohort	Canada	V: 85,670 UV: 56,336	V: 32.3 ± 4.5 UV: 30.8 ± 5.3	Moderna mRNA-1273, Pfizer-BioNTech BNT162b2, Mixed doses	2021.05.01–2022.09.02	Neonatal death, maternal infection, NICU, preterm birth	8

Raanan Meyer et al. (2023)	Retrospective cohort	Israel	V: 2071 UV: 1030	NA	mRNA vaccine	2021.02.09–2021.06.02	Maternal infection	9

Peretz-Machluf et al. (2022)	Retrospective cohort	Israel	V: 3240 UV: 460	V: 32.51 ± 5.07 UV: 31.99 ± 5.51	Pfizer-BioNTech BNT162b2	2021.03–2021.07	PPROM premature rupture of membranes/placental abruption, NICU, preterm birth	9

Theiler et al. (2021)	Retrospective cohort	USA	V: 140 UV: 1862	V: 31.8 ± 3.7 UV: 30.5 ± 5.2	Moderna mRNA-1273, Pfizer-BioNTech BNT162b2, adenovirus vaccine	2020.12.10–2021.04.19	Maternal infection, NICU, maternal hospitalization, postpartum hemorrhage, neonatal death, preterm birth	7

Boelig et al. (2022)	Retrospective cohort	USA	V: 49 UV: 198	V: 34.1 ± 4.5 UV: 30.8 ± 5.5	mRNA vaccine	2021.03–2021.07	Maternal vascular hypoperfusion, preterm birth	7

Zerbo et al. (2023)	Retrospective cohort	USA	V: 16,242 UV: 41,446	NA	mRNA vaccine	2020.12.15–2022.09.30	Maternal hospitalization	9

Zerbo et al. (2023) (2)	Case–control	USA	V: 10,893 UV: 19,418	V: 32.59 ± 4.30 UV: 31.08 ± 4.77	mRNA vaccine	2020.12.15–2022.05.31	Maternal hospitalization	9

Goh et al. (2023)	Retrospective cohort	Singapore	V: 7120 UV: 172	NA	mRNA vaccine	2022.01.01–2022.09.30	Neonatal/infant infection	8

Kugelman et al. (2023)	Retrospective cohort	Israel	V: 930 UV: 964	V: 32 ± 3 UV: 31 ± 3.5	Pfizer-BioNTech BNT162b2	2021.02.01–2021.07.31	Intrauterine fetal death, NICU, preterm birth	8

Dagan et al. (2021)	Observational cohort	Israel	V: 10,861 UV: 10,861	V: 30 ± 3.5 UV: 30 ± 3.5	Pfizer-BioNTech BNT162b2	2020.12.20–2021.06.03	Maternal infection, maternal hospitalization, severe or critical maternal COVID-19	9

Changizi et al. (2023)	Retrospective cohort	Iran	V: 19,922 UV: 70,147	V: 28.6 UV: 28.6	Sinopharm Biotech, adenovirus vaccine	2021.09.01–2022.01.30	Stillbirth, neonatal death, maternal hospitalization, maternal ICU admission/death	7

Halasa et al. (2022)	Test-negative case–control	USA	V: 234 UV: 815	NA	mRNA vaccine	2021.07.01–2022.03.08	Neonatal death, NICU, neonatal/infant hospitalization, preterm birth	8

Minghui et al. (2023)	Prospective cohort	China	V: 93 UV: 160	V: 31.65 ± 4.36 UV: 31.98 ± 4.01	Inactivated vaccine	2021.03.01–2022.02.02	Postpartum hemorrhage, PPROM premature rupture of membranes/placental abruption, preterm birth	6

Zare Sakhvidi et al. (2023)	Retrospective cohort	Iran	V: 720 UV: 502	V: 29.3 ± 5.9 UV: NA	Inactivated vaccine	2021.08.01–2022.01.01	Preterm birth	8

Rottenstreich et al. (2023)	Multicenter retrospective cohort	Israel	V: 1094 UV: 863	V: 28.9 ± 5.7 UV: 28.4 ± 6.2	mRNA vaccine	2021.08–2021.11	Postpartum hemorrhage, intrauterine fetal death, chorioamnionitis, maternal ICU admission/death	9

Rottenstreich et al. (2022)	Retrospective cohort	Israel	V: 712 UV: 1063	V: 30.6 ± 5.8 UV: 31.98 ± 4.01	mRNA vaccine	2021.01.19–2021.04.27	NICU, postpartum hemorrhage, intrauterine fetal death	9

Lipschuetz et al. (2022)	Retrospective cohort	Israel	V: 35,595 UV: 13,273	NA	mRNA vaccine	2021.08.24–2022.03.15	NICU, neonatal/infant hospitalization, preterm birth	9

Magnus et al. (2022) (N)	Registry-based retrospective cohort	Norway	V: 7590 UV: 46,522	V: 31.4 ± 4.5 UV: 30.7 ± 4.7	Moderna mRNA-1273, Pfizer-BioNTech BNT162b2, adenovirus vaccine	2021.01.01–2022.01.15	Stillbirth, maternal infection, NICU, preterm birth	9

Magnus et al. (2022) (S)	Registry-based retrospective cohort	Sweden	V: 28,506 UV: 129,015	V: 32.2 ± 4.6 UV: 30.5 ± 4.8	Moderna mRNA-1273, Pfizer-BioNTech BNT162b2, adenovirus vaccine	2021.01.01–2022.01.12	Stillbirth, maternal infection, NICU, preterm birth	9

Magnus et al. (2021)	Case–control	Norway	V: 1003 UV: 17,474	NA	Moderna mRNA-1273, Pfizer-BioNTech BNT162b2, adenovirus vaccine	2021.08.15–2021.02.15	Spontaneous abortion	8

Lu et al. (2023)	Prospective cohort	China	V: 497 UV: 348	V: 30.03 ± 3.43 UV: 30.14 ± 3.80	Inactivated vaccine	2021.07–2021.10	NICU, maternal ICU admission/death, postpartum hemorrhage, PPROM premature rupture of membranes/placental abruption, preterm birth	8

Lindsay et al. (2023)	Cohort	Scotland	V: 11,202 UV: 22,404	V: 32 UV: 31	Moderna mRNA-1273, Pfizer-BioNTech BNT162b2, mixed doses	2020.11.08–2021.06.02	Maternal ICU admission/death, stillbirth, preterm birth	9

Hui et al. (2023)	Multicentric cohort	Australia	V: 9682 UV: 2607	NA	mRNA vaccine	2021.02.11–2021.05.31	Stillbirth, preterm birth, postpartum hemorrhage	8

Hui et al.(2023)	Multicentric cohort	Australia	V: 9667 UV: 2586	NA	mRNA vaccine	2021.02.11–2021.05.31	Maternal infection, NICU	8

Ghesquiere et al. (2023)	Prospective cohort	Canada	V: 597 UV: 191	V: 30.03 ± 3.43 UV: 30.14 ± 3.80	Lacking detailed information	2021.12–2021.12	Maternal infection, preterm birth	9

Law et al. (2023)	Case–control	China	V: 233 UV: 229	V: 31.9 UV: 31.26	mRNA vaccine	2021.07.01–2021.10.31	Postpartum hemorrhage, stillbirth, PPROM premature rupture of membranes/placental abruption, placental previa, NICU	6

Yland et al. (2023)	Internet-based, prospective preconception cohort	Canada	V: 1356 UV: 455	V: 31.3 UV: 30.6	Adenovirus vaccine	2020.11.20–2022.11.23	Spontaneous abortion	7

Morgan et al. (2023)	Retrospective cohort	USA	V: 2106 UV: 14,026	V: 31.56 ± 5.15 UV: 27.83 ± 5.8	mRNA vaccine	2021.01.01–2021.12.31	Neonatal death, maternal infection, NICU, severe or critical maternal COVID-19, stillbirth, preterm birth	8

Morgan et al. (2022)	Retrospective cohort	USA	V: 1332 UV: 8760	V: 32.1 ± 5.9 UV: 27.8 ± 4.9	Moderna mRNA-1273, Pfizer-BioNTech BNT162b2, adenovirus vaccine	2021.06.15–2021.08.20	Stillbirth, maternal infection, severe or critical maternal COVID-19	8

Citu et al. (2022)	Observational retrospective	Germany	V: 927 UV: 2167	V: 29.5 ± 7.3 UV: 31.6 ± 5.6	Moderna mRNA-1273, Pfizer-BioNTech BNT162b2, adenovirus vaccine	2020.01–2022.01	Spontaneous abortion	8

Goldshtein et al. (2022)	Retrospective cohort	Israel	V: 16,697 UV: 7591	V: 31.2 ± 5.3 UV: 31.1 ± 5.6	mRNA vaccine	2021.03–2021.10.31	Neonatal/infant hospitalization, preterm birth	9

Goldshtein et al. (2021)	Retrospective cohort	Israel	V: 7530 UV: 7530	V: 31.1 ± 5.01 UV: 31.0 ± 4.85	mRNA vaccine	2020.12.19–2021.02.28	Spontaneous abortion, stillbirth, maternal infection, maternal hospitalization, preterm birth	7

Barros et al. (2024)	Prospective cohort	18 countries	V: 914 UV: 631	V (1 dose): 30.1 ± 6.2 V (2 doses) :31.1 ± 5.7 V (3 doses): 33.7 ± 5.2 UV: 30.6 ± 6.1	Moderna, Pfizer-BioNTech, adenovirus vaccine, inactivated vaccine	2021.09.10–2022.06.23	Preterm birth, premature rupture of membranes, NICU, infant infection	8

Cai et al. (2024)	Retrospective cohort	China	V: 603 UV: 603	V: 30.58 ± 4.62 UV: 30.43 ± 4.29	Inactivated vaccine	2022.01–2022.06	Preterm birth, premature rupture of membranes, placenta previa or placenta implantation, placental abruption, chorioamnionitis, postpartum hemorrhage, infant hospitalization	9

De Virgilio Suglia et al. (2024)	Retrospective cohort	Italy	V: 1657 UV: 1689	NA	Moderna, Pfizer-BioNTech, adenovirus vaccine	2021.02.22–2022.12.31	Infant infection	8

Denoble et al. (2024)	Case–control	USA	V: 429 UV: 669	V: 32.4 ± 4.9 UV: 30.7 ± 5.8	Moderna, Pfizer-BioNTech, adenovirus vaccine	2021.01.13–2022.02.28	Stillbirth	8

Faherty et al. (2024)	Retrospective cohort	USA	V: 45,296 UV: 250,164	V (before Pregnancy): 31 (28–34) V (during Pregnancy): 31 (27–34) UV: 29 (25–33)	Moderna, Pfizer-BioNTech, adenovirus vaccine	2020.12.10–2023.10.12	Preterm birth, stillbirth	8

Hatami et al. (2024)	Retrospective cohort	Iran	V: 17,485 UV: 112,003	V: 31.33(5.75) UV: 31.16(5.67)	Sinopharm biotech	2021.03.21–2022.03.21	Preterm birth, stillbirth, NICU	8

Iannaccone et al. (2024)	Prospective cohort	Germany	V: 2156 UV: 5174	NA	NA	2020.04.03–2022.12.31	Preterm birth, stillbirth	7

Mahyuddin et al. (2024)	Prospective observational	Singapore	V: 98 UV: 0	NA	BNT162b2, mRNA-1273, Sinopharm BBIBP-CorV	2021.08.24–2022.05.24	Maternal infection	8

Mensah et al. (2024)	Retrospective cohort, matched case–control	England	V: 182,582 UV: 331,431	NA	BNT162b2, mRNA-1273	2021.04–2022.03	Preterm birth, neonatal death, stillbirth, mother ICU occupancy rate, maternal infection	9

Norman et al. (2024)	Retrospective cohort, matched case–control	Sweden and Norway	V: 94,303 UV: 102,167	NA	BNT162b2, mRNA-1273, ChAdOx1-S	2021.06–2023.01	Preterm birth, neonatal death, NICU	9

Santos et al. (2024)	Retrospective cohort	USA	V: 1158 UV: 938	V: 31.4 ± 4.3 UV: 29.1 ± 5.3	BNT162b2, mRNA-1273	2020.12.10–2021.12.31	Neonatal death, stillbirth, maternal infection	8

Arun Suseeladevi et al. (2024)	England-wide cohort	England	V: 53,060 UV: 133,405	V: 31.1 ± 5.1 UV: 28.7 ± 5.4	BNT162b2, ChAdOx1-S	2021.05.25–2022.10.28	Preterm birth, stillbirth	9

Velez et al. (2024)	Population-based cohort	Ontario, Canada	V: 83,475 UV: 162,784	V: 31.2 ± 5.4, 31.0 ± 5.1 UV: 30.6 ± 5.5	mRNA vaccine, adenovirus-vectored vaccine	2020.12.14–2021.12.01	Spontaneous abortion	9

Yeniocak et al. (2024)	Retrospective cohort	Turkey	V: 12 UV: 297	V: 30.58 ± 5.66, UV: 29.0 ± 5.52	NA	2020.10–2022.07	Mother ICU occupancy rate	8

Zels et al. (2024)	Retrospective	Belgium	V: 127 UV: 55	NA	NA	2020.01–2022.08	Intrauterine fetal death, MVM, chorioamnionitis	7

Zhang et al. (2024)	Single-center retrospective cohort	China	V: 369 UV: 231	V: 29.72 ± 5.30 UV: 30.09 ± 5.26	Adenovirus type 5 vector, vero cell, CHO cell	2020.01.01–2022.09.09	Spontaneous abortion	7

**Table 2:** Overall and subgroup meta-analyses of maternal outcomes.

	Study *N*	OR (95% CI)	*p* value	*I* ^2^	Subgroup heterogeneity
SARS‐CoV‐2 infection in mothers	24	0.5226 [0.4528–0.6031]	**< 0.0001**	96.5%	
By vaccine types					*p*=0.3266
mRNA vaccine	9	0.4794 [0.3437–0.6686]		94.6%	
Inactivated vaccine	1	0.5706 [0.5148–0.6326]		0.00%	
By number of doses					**p**=0.0114
1 dose	1	0.7692 [0.6714–0.8811]		0.00%	
2 dose	5	0.3965 [0.2417–0.6503]		94.5%	
Maternal hospitalization	8	0.5842 [0.4528–0.7537]	**< 0.0001**	79.3%	
By vaccine types					**p** < 0.0001
Inactivated vaccine	1	11.1895 [10.5282–11.8923]		0.00%	
mRNA vaccine	4	0.1954 [0.0641–0.5956]		80.6%	
Adenovirus vaccine	1	10.9695 [8.6784–13.8655]		0.00%	
Severe/critical maternal outcomes	11	0.4952 [0.3285–0.7464]	**0.0008**	91.0%	
By vaccine types					*p*=0.6375
mRNA vaccine	5	0.7860 [0.6231–0.9914]		65.8%	
Inactivated vaccine	1	1.0003 [0.3770–2.6539]		0.00%	

*Note:* The bold values indicate analyses detecting significant differences.

**Table 3:** Overall and subgroup meta-analyses of neonatal outcomes.

	Study *N*	OR (95% CI)	*I* ^2^	Subgroup heterogeneity
SARS‐CoV‐2 infection in infants	7	0.6895 [0.4975–0.9556]	82.4%	
By vaccine types				*p*=0.2722
mRNA vaccine	2	0.8037 [0.4883; 1.3228]	86.2%	
Inactivated vaccine	5	0.5941 [0.4834; 0.7303]	14.1%	
By variants				**p** < 0.0001
Delta	3	0.2033 [0.1304–0.3170]	39.1%	
Omicron	3	0.9649 [0.6935–1.3425]	86.6%	
Neonatal hospitalization	6	0.6896 [0.5451–0.8724]	77.4%	
NICU/PICU	27	0.9110 [0.8436–0.9837]	89.3%	
By vaccine types				**p**=0.0394
mRNA vaccine	11	0.8241 [0.6951–0.9771]	94.8%	
Inactivated vaccine	5	0.9938 [0.9433–1.0469]	0.00%	
By trimester				*p*=0.5578
1^st^ trimester	7	1.0836 [0.7827–1.5002]	90.7%	
2^nd^ trimester	5	1.0269 [0.8422–1.2521]	95.9%	
3^rd^ trimester	7	0.9443 [0.8772–0.0165]	57.7%	
By number of doses				**p**=0.0427
1 dose	6	1.0352 [0.9392–1.1411]	74.8%	
2 doses	8	0.9072 [0.8354–0.9852]	78.2%	

*Note:* The bold values indicate analyses detecting significant subgroup differences.

**Table 4:** Overall and subgroup meta-analyses of preterm birth.

	Study *N*	OR (95% CI)	*I* ^2^	Subgroup heterogeneity
Preterm birth	45	0.8695 [0.8106–0.9328]	93.6%	
By prior infection status				**p**=0.0371
Possible prior infection	25	0.8182 [0.7402–0.9043]	96.2%	
No prior infection	20	0.9349 [0.8668–1.0084]	66.3%	
By vaccine types				**p**=0.0263
mRNA vaccine	18	0.9902 [0.6983; 1.4040]	99.8%	
Inactivated vaccine	11	0.9538 [0.7856–1.1581]	60.9%	
Adenovirus vaccine	2	1.5362 [1.1433; 2.0641]	98.6%	
By trimester				**p**=0.0003
1^st^ trimester	10	1.0395 [0.9258–1.1672]	79.8%	
2^nd^ trimester	9	1.1361 [0.9770–1.3211]	95.7%	
3^rd^ trimester	9	0.7596 [0.6569–0.8784]	92.8%	
By number of doses				**p** < 0.0001
1 dose	7	1.1440 [1.0139–1.2908]	56.6%	
2 dose	12	0.8027 [0.7137–0.9029]	84.0%	
By type of preterm birth				*p*=0.9671
Spontaneous	7	0.7625 [0.6729–0.8642]	85.1%	
Provider-initiated	6	0.7578 [0.5776–0.9941]	94.2%	

*Note:* The bold values indicate analyses detecting significant subgroup differences.

**Table 5:** Overall and subgroup meta-analyses of other obstetric outcomes.

	Study *N*	OR (95% CI)	*I* ^2^	Subgroup heterogeneity
Abortion	17	1.0184 [0.8938–1.1603]	93.0%	
By prior infection status				*p*=0.3969
Possible prior infection	8	0.9597 [0.8132–1.1325]	90.3%	
No prior infection	7	1.0716 [0.8825–1.3011]	92.7%	
By type of abortion				*p*=0.4117
Spontaneous abortion	15	1.0060 [0.8806–1.1493]	93.8%	
Threatened abortion	2	1.4137 [0.6345–3.1499]	43.8%	
By number of doses				*p*=0.0994
1 dose	3	1.0113 [0.9360–1.0927]	0.00%	
2 dose	3	0.7613 [0.5479–1.0577]	70.7%	
By vaccine types				*p*=0.6989
mRNA vaccine	5	1.0074 [0.9424–1.0769]	25.5%	
Inactivated vaccine	3	0.9906 [0.7425–1.3215]	0.00%	
Adenovirus vaccine	3	2.0130 [0.3993–10.1494]	99.6%	
Postpartum hemorrhage	19	0.9897 [0.4338–2.2576]	99.5%	
By prior infection status				*p*=0.3078
Possible prior infection	9	0.8563 [0.6747–1.0867]	53.6%	
No prior infection	8	1.0047 [0.8275–1.2199]	33.3%	
By vaccine types				**p**=0.0435
mRNA vaccine	7	1.0342 [0.8954–1.1945]	29.3%	
Inactivated vaccine	5	0.4881 [0.2389–0.9974]	56.8%	
By trimester				*p*=0.9065
1^st^ trimester	1	1.0712 [0.5740–1.9989]	0.00%	
2^nd^ trimester	1	1.1625 [0.7871–1.7168]	0.00%	
3^rd^ trimester	1	1.0317 [0.7190–1.4806]	0.00%	
Intrauterine fetal death	10	0.7617 [0.5344–1.0856]	26.0%	
By prior infection status				*p*=0.1653
Possible prior infection	3	0.4338 [0.1587–1.1860]	43.1%	
No prior infection	7	0.9084 [0.6865–1.2019]	0.00%	
By vaccine types				*p*=0.5371
mRNA vaccine	5	0.9145 [0.6885–1.2147]	0.00%	
Inactivated vaccine	1	0.3320 [0.0135–8.1893]	0.00%	
By trimester				*p*=0.7043
1^st^ trimester	1	0.3320 [0.0135–8.1893]	0.00%	
2^nd^ trimester	1	1.0419 [0.5116–2.1216]	0.00%	
3^rd^ trimester	2	0.6453 [0.1339–3.1112]	73.5%	
By race				*p*=0.1393
White	1	2.0419 [0.4901–8.5066]	0.00%	
Black	1	4.5267 [0.6134–33.4041]	0.00%	
Asian	2	0.4418 [0.1014–1.9250]	0.00%	
By number of doses				*p*=0.9131
1 dose	1	0.6190 [0.0250–15.3046]	0.00%	
2 dose	3	0.7412 [0.4859–1.1307]	0.00%	
Neonatal death	10	0.4739 [0.2628–0.8545]	95.2%	
By prior infection status				*p*=0.6052
Possible prior infection	5	0.5559 [0.2826–1.0934]	81.0%	
No prior infection	5	0.4309 [0.2165–0.8578]	94.7%	
Stillbirth	22	0.6359 [0.5314–0.7609]	70.1%	
By prior infection status				*p*=0.5571
Possible prior infection	15	0.6175 [0.5006–0.7617]	72.7%	
No prior infection	7	0.7055 [0.4767–1.0440]	61.8%	
By vaccine types				*p*=0.4804
mRNA vaccine	9	0.3551 [0.1215–1.0379]	98.3%	
Inactivated vaccine	2	1.9125 [0.1277–28.6341]	68.2%	
Adenovirus vaccine	2	0.2114 [0.0078–5.6986]	99.6%	
By trimester				*p*=0.1064
1^st^ trimester	2	1.0494 [0.8717–1.2633]	0.0%	
2^nd^ trimester	2	0.9063 [0.7975–1.0298]	0.0%	
3^rd^ trimester	2	0.5317 [0.2734–1.0341]	84.5%	
Premature rupture of membranes	9	0.9425 [0.6861–1.2946]	70.4%	
By prior infection status				*p*=0.5609
Possible prior infection	4	0.8440 [0.4059–1.7550]		
No prior infection	5	1.0577 [0.8602–1.3005]		
By vaccine types				*p*=0.7815
mRNA vaccine	3	0.8762 [0.5584–1.3747]	0.0%	
Inactivated vaccine	4	0.9759 [0.5280–1.8038]	87.5%	
By trimester				*p*=0.9266
1^st^ trimester	2	1.5050 [0.4897–4.6247]	65.4%	
2^nd^ trimester	1	1.1406 [0.2909–4.4726]	0.00%	
3^rd^ trimester	1	1.6222 [0.4667–5.6388]	0.00%	
By number of doses				*p*=0.5482
1 dose	2	1.0707 [0.7515–1.5255]	0.00%	
2 dose	2	0.9347 [0.7158–1.2206]	0.00%	
Abnormality of placenta	14	0.8645 [0.6371–1.1731]	27.9%	
By prior infection status				*p*=0.5399
Possible prior infection	4	0.5661 [0.1709–1.8760]	73.3%	
No prior infection	10	0.8330 [0.6179–1.1228]	0.0%	
By type of abnormality of placenta				*p*=0.7472
Combination	1	0.9037 [0.5515–1.4808]	0.00%	
Placental previa	4	0.5320 [0.2312–1.2239]	0.00%	
Placental abruption	3	0.8869 [0.4105–1.9164]	0.00%	
Placental pathology	6	0.7920 [0.4449–1.4097]	62.5%	
MVM (maternal vascular malperfusion)	4	0.5079 [0.2453–1.0516]	21.3%	
Chorioamnionitis	5	1.3460 [0.9408–1.9258]	15.8%	

*Note:* The bold values indicate analyses detecting significant subgroup differences.

## Data Availability

The data underlying this article are available in the article and its online supporting information.

## Supporting Information

Search strategy, supporting figures (described as Figures S1–S21 in the article), and NOS are provided in the supporting information file. Supporting Figure 1: Forest plots showing the results of meta-analyses for maternal infection (booster vs. 2 doses). Supporting Figure 2: Forest plots showing the results of meta-analyses for maternal infection (vaccinated during pregnancy vaccinated before pregnancy and unvaccinated). Supporting Figure 3: Forest plots showing the results of meta-analyses for maternal hospitalization (booster vs. unvaccinated). Supporting Figure 4: Forest plots showing the results of meta-analyses for maternal hospitalization (Delta waves). Supporting Figure 5: Forest plots showing the results of meta-analyses for maternal hospitalization (Omicron waves). Supporting Figure 6: Forest plots showing the results of meta-analyses for maternal hospitalization (Booster in Delta waves). Supporting Figure 7: Forest plots showing the results of meta-analyses for maternal hospitalization (Booster in Omicron waves). Supporting Figure 8: Forest plots showing the results of meta-analyses for maternal hospitalization (2 doses). Supporting Figure 9: Forest plots showing the results of meta-analyses for NICU (booster vs. unvaccinated). Supporting Figure 10: Forest plots showing the results of meta-analyses for neonatal infection (2 doses). Supporting Figure 11: Forest plots showing the results of meta-analyses for neonatal infection (booster vs. unvaccinated). Supporting Figure 12: Forest plots showing the results of meta-analyses for neonatal hospitalization (mRNA vaccine). Supporting Figure 13: Forest plots showing the results of meta-analyses for neonatal hospitalization (1st trimester). Supporting Figure 14: Forest plots showing the results of meta-analyses for neonatal hospitalization (2 doses). Supporting Figure 15: Forest plots showing the results of meta-analyses for stillbirth (booster vs. unvaccinated). Supporting Figure 16: Forest plots showing the results of meta-analyses for preterm (booster vs. unvaccinated). Supporting Figure 17: Forest plots showing the results of meta-analyses for preterm (< 34 weeks). Supporting Figure 18: Forest plots showing the results of meta-analyses for intrauterine fetal death (booster vs. unvaccinated). Supporting Figure 19: Forest plots showing the results of meta-analyses for postpartum hemorrhage (booster vs. unvaccinated). Supporting Figure 20: Forest plots showing the results of meta-analyses for abortion (vaccinated in 1st trimester vs. unvaccinated). Supporting Figure 21: Forest plots showing the results of meta-analyses for abortion (vaccinated during pregnancy vs. vaccinated before pregnancy and unvaccinated). Supporting Table 1: Quality evaluation of the eligible cohort studies with Newcastle–Ottawa scale. Supporting Table 2: Quality evaluation of the eligible case–control studies with Newcastle–Ottawa scale.

## References

[1] Metz T. D., Clifton R. G., Hughes B. L. (2022). Association of SARS-CoV-2 Infection With Serious Maternal Morbidity and Mortality From Obstetric Complications. *JAMA*.

[2] Allotey J., Stallings E., Bonet M. (2020). Clinical Manifestations, Risk Factors, and Maternal and Perinatal Outcomes of Coronavirus Disease 2019 in Pregnancy: Living Systematic Review and Meta-Analysis. *BMJ*.

[3] Rasmussen S. A., Jamieson D. J. (2022). COVID-19 and Pregnancy. *Infectious Disease Clinics of North America*.

[4] Di Mascio D., Khalil A., Saccone G. (2020). Outcome of Coronavirus Spectrum Infections (SARS, MERS, COVID-19) during Pregnancy: a Systematic Review and Meta-Analysis. *American Journal of Obstetrics & Gynecology MFM*.

[5] Male V. (2022). SARS-CoV-2 Infection and COVID-19 Vaccination in Pregnancy. *Nature Reviews Immunology*.

[6] (2022). Update to Living Systematic Review on Covid-19 in Pregnancy. *BMJ*.

[7] Lopez Bernal J., Andrews N., Gower C. (2021). Effectiveness of the Pfizer-BioNTech and Oxford-AstraZeneca Vaccines on Covid-19 Related Symptoms, Hospital Admissions, and Mortality in Older Adults in England: Test Negative Case-Control Study. *BMJ*.

[8] Moreira E. D., Kitchin N., Xu X. (2022). Safety and Efficacy of a Third Dose of BNT162b2 Covid-19 Vaccine. *New England Journal of Medicine*.

[9] Thomas S. J., Moreira E. D., Kitchin N. (2021). Safety and Efficacy of the BNT162b2 mRNA Covid-19 Vaccine through 6 Months. *New England Journal of Medicine*.

[10] Feikin D. R., Higdon M. M., Abu-Raddad L. J. (2022). Duration of Effectiveness of Vaccines against SARS-CoV-2 Infection and COVID-19 Disease: Results of a Systematic Review and Meta-Regression. *The Lancet*.

[11] Feldstein L. R., Britton A., Grant L. (2024). Effectiveness of Bivalent mRNA COVID-19 Vaccines in Preventing SARS-CoV-2 Infection in Children and Adolescents Aged 5 to 17 Years. *JAMA*.

[12] Tregoning J. S., Flight K. E., Higham S. L., Wang Z., Pierce B. F. (2021). Progress of the COVID-19 Vaccine Effort: Viruses, Vaccines and Variants versus Efficacy, Effectiveness and Escape. *Nature Reviews Immunology*.

[13] Piechotta V., Siemens W., Thielemann I. (2023). Safety and Effectiveness of Vaccines against COVID-19 in Children Aged 5-11 Years: a Systematic Review and Meta-Analysis. *The Lancet Child & Adolescent Health*.

[14] Marwa M. M., Kinuthia J., Larsen A. (2023). COVID-19 Vaccine Hesitancy Among Pregnant and Postpartum Kenyan Women. *International Journal of Gynecology & Obstetrics*.

[15] Stang A. (2010). Critical Evaluation of the Newcastle-Ottawa Scale for the Assessment of the Quality of Nonrandomized Studies in Meta-Analyses. *European Journal of Epidemiology*.

[16] Salomoni M. G., Di Valerio Z., Gabrielli E. (2021). Hesitant or Not Hesitant? A Systematic Review on Global COVID-19 Vaccine Acceptance in Different Populations. *Vaccines*.

[17] Mekuriaw B. Y., Nigatu D., Dessie A. M., Asresie M. B. (2023). Intention to Take COVID-19 Vaccine and Associated Factors Among Pregnant Women Attending Antenatal Care at Public Health Facilities in Bahir Dar City, Northwest Ethiopia. *BMC Women’s Health*.

[18] McClure C. C., Cataldi J. R., O’Leary S. T. (2017). Vaccine Hesitancy: Where We Are and where We Are Going. *Clinical Therapeutics*.

[19] Calvert C., Carruthers J., Denny C. (2022). A Population-Based Matched Cohort Study of Early Pregnancy Outcomes Following COVID-19 Vaccination and SARS-CoV-2 Infection. *Nature Communications*.

[20] Yland J. J., Wesselink A. K., Regan A. K. (2023). A Prospective Cohort Study of Preconception COVID-19 Vaccination and Miscarriage. *Human Reproduction*.

[21] Teng Z., Qian M., Wei W., Lizhou S., Meilin L. (2023). Acute Adverse Events and Pregnancy Outcome after Inactivated COVID-19 Vaccination in First Trimester. *Chinese Journal of Preventive Medicine*.

[22] Goldshtein I., Nevo D., Steinberg D. M. (2021). Association between BNT162b2 Vaccination and Incidence of SARS-CoV-2 Infection in Pregnant Women. *JAMA*.

[23] Goldshtein I., Steinberg D. M., Kuint J. (2022). Association of BNT162b2 COVID-19 Vaccination during Pregnancy with Neonatal and Early Infant Outcomes. *JAMA Pediatrics*.

[24] Carlsen E. Ø., Magnus M. C., Oakley L. (2022). Association of COVID-19 Vaccination during Pregnancy with Incidence of SARS-CoV-2 Infection in Infants. *JAMA Internal Medicine*.

[25] Fell D. B., Dhinsa T., Alton G. D. (2022). Association of COVID-19 Vaccination in Pregnancy With Adverse Peripartum Outcomes. *JAMA*.

[26] Chen Z., Mu X., Wang X. (2023). Association of Maternal Inactivated COVID-19 Vaccination within 3 Months before Conception with Neonatal Outcomes. *Vaccines*.

[27] Magnus M. C., Örtqvist A. K., Dahlqwist E. (2022). Association of SARS-CoV-2 Vaccination During Pregnancy With Pregnancy Outcomes. *JAMA*.

[28] Meyer R., Mohr-Sasson A., Mashiach R., Levin G. (2023). BNT162b2 COVID-19 Vaccine and Gynecologic Emergency Department Visit Diagnoses—A Study from a Large Tertiary Center. *International Journal of Gynaecology & Obstetrics: The Official Organ of the International Federation of Gynaecology and Obstetrics*.

[29] Rottenstreich M., Rotem R., Wiener-Well Y., Grisaru-Granovsky S., Sela H. Y. (2022). Covid-19 Third Vaccination during Pregnancy: Maternal and Neonatal Outcomes—A Retrospective Study. *Archives of Gynecology and Obstetrics*.

[30] Suseeladevi A. K., Denholm R., Retford M. (2024). COVID-19 Vaccination and Birth Outcomes of 186,990 Women Vaccinated before Pregnancy: an England-wide Cohort Study. *The Lancet Regional Health-Europe*.

[31] Blakeway H., Prasad S., Kalafat E. (2022). COVID-19 Vaccination during Pregnancy: Coverage and Safety. *American Journal of Obstetrics and Gynecology*.

[32] Rottenstreich M., Sela H. Y., Rotem R., Kadish E., Wiener‐Well Y., Grisaru‐Granovsky S. (2022). Covid-19 Vaccination during the Third Trimester of Pregnancy: Rate of Vaccination and Maternal and Neonatal Outcomes, a Multicentre Retrospective Cohort Study. *BJOG: An International Journal of Obstetrics & Gynaecology*.

[33] Zels G., Colpaert C., Leenaerts D. (2024). COVID-19 Vaccination Protects Infected Pregnant Women from Developing SARS-CoV-2 Placentitis and Decreases the Risk for Stillbirth. *Placenta*.

[34] Zare Sakhvidi M., Lotfi M. H., Fallahzadeh H., Hosseini S., Kalantari F., Taheri Soodejani M. (2023). COVID-19 Vaccine in Pregnant Women and Pregnancy Outcomes: A Historical Cohort in Center of Iran. *Women’s Health*.

[35] Gray K. J., Bordt E. A., Atyeo C. (2021). COVID-19 Vaccine Response in Pregnant and Lactating Women: a Cohort Study.

[36] Mensah A., Stowe J., Jardine J. (2024). COVID-19 Vaccine Safety in Pregnancy, A Nested Case–Control Study in Births from April 2021 to March 2022, England. *BJOG: An International Journal of Obstetrics and Gynaecology*.

[37] Piekos S. N., Hwang Y. M., Roper R. T. (2023). Effect of COVID-19 Vaccination and Booster on Maternal–Fetal Outcomes: a Retrospective Cohort Study. *The Lancet Digital Health*.

[38] Gwak E., Kim T., Shin J. (2023). Effectiveness and Safety of COVID-19 Vaccination during Preconceptional and Preclinical Pregnancy Period: A National Population Study. *Journal of Korean Medical Science*.

[39] Guedalia J., Lipschuetz M., Calderon-Margalit R. (2022). Effectiveness of a Third BNT162b2 mRNA COVID-19 Vaccination during Pregnancy: a National Observational Study in Israel. *Nature Communications*.

[40] Zerbo O., Ray G. T., Fireman B. (2023). Effectiveness of COVID-19 Vaccination during Pregnancy by Circulating Viral Variant. *AJOG Global Reports*.

[41] Dagan N., Barda N., Biron-Shental T. (2021). Effectiveness of the BNT162b2 mRNA COVID-19 Vaccine in Pregnancy. *Nature Medicine*.

[42] Tripathy G. S., Rath T. S., Behera S., Lekha K. S., Kar D., Pendyala S. (2023). Effects of Covid-19 Vaccination During Pregnancy on the Obstetric and Neonatal Outcomes in A Tertiary Health Care Center. *Journal of Mother and Child*.

[43] De Virgilio Suglia C., Stefanizzi P., Graziano G. (2024). Efficacy of Vaccination during Pregnancy in Reducing the Risk of SARS-CoV-2 Infection in Infants Younger Than 12 Months. Puglia (Italy), 2021–23. *Human Vaccines & Immunotherapeutics*.

[44] Ghesquiere L., Boivin G., Demuth B. (2023). Impact of COVID-19 and Vaccination during Pregnancy on Placenta-Mediated Complications (COVIGRO Study). *Journal of Obstetrics and Gynaecology Canada*.

[45] Boelig R. C., Aghai Z. H., Chaudhury S., Kazan A. S., Chan J. S., Bergmann-Leitner E. (2022). Impact of COVID-19 Disease and COVID-19 Vaccination on Maternal or Fetal Inflammatory Response, Placental Pathology, and Perinatal Outcomes. *American Journal of Obstetrics and Gynecology*.

[46] Jarraya A., Kammoun M., Amouri S. (2023). Impact of COVID-19 Vaccination Among Pregnant Women Requiring Hospital Admission: Prospective Observational Research. *Italian Journal of Gynaecology and Obstetrics*.

[47] Ibroci E., Liu X., Lieb W. (2023). Impact of Prenatal COVID-19 Vaccination on Delivery and Neonatal Outcomes: Results from a New York City Cohort. *Vaccine*.

[48] Yeniocak A. S., Tercan C., Dagdeviren E., Arabaci O., Arabaci E. (2024). Impact of SARS-CoV-2 Infection and Vaccination on Cesarean Section Outcomes: a Retrospective Analysis. *Annals of Saudi Medicine*.

[49] Kim H., Kim H. S., Kim H. M. (2023). Impact of Vaccination and the Omicron Variant on COVID-19 Severity in Pregnant Women. *American Journal of Infection Control*.

[50] Yang C., Zheng Z., Zheng P. (2023). Inactivated COVID-19 Vaccines in Peri-Pregnancy Period: Evaluation of Safety for Both Pregnant Women and Neonates. *Vaccine*.

[51] Li M., Hao J., Jiang T. (2023). Maternal and Neonatal Safety of COVID-19 Vaccination during the Peri-Pregnancy Period: A Prospective Study. *Journal of Medical Virology*.

[52] Süt H., Aynaoğlu Yıldız G., Şeker E., Ümit C., Koçar M., Koç A. (2023). Maternal and Perinatal Outcomes of COVID-19 Vaccination during Pregnancy. *Journal of the Turkish-German Gynecological Association*.

[53] Santos J., Miller M., Branda M. E., Mehta R. A., Theiler R. N. (2024). Maternal COVID-19 Vaccination Status and Association with Neonatal Congenital Anomalies. *Front Pediatr*.

[54] Morgan J. A., Biggio J. R., Martin J. K. (2022). Maternal Outcomes after Severe Acute Respiratory Syndrome Coronavirus 2 (SARS-CoV-2) Infection in Vaccinated Compared with Unvaccinated Pregnant Patients. *Obstetrics & Gynecology*.

[55] Lipschuetz M., Guedalia J., Cohen S. M. (2023). Maternal Third Dose of BNT162b2 mRNA Vaccine and Risk of Infant COVID-19 Hospitalization. *Nature Medicine*.

[56] Barros F. C., Gunier R. B., Rego A. (2024). Maternal Vaccination against COVID-19 and Neonatal Outcomes during Omicron: INTERCOVID-2022 Study. *American Journal of Obstetrics and Gynecology*.

[57] Velez M. P., Fell D. B., Shellenberger J. P., Kwong J. C., Ray J. G. (2024). Miscarriage after SARS-CoV-2 Vaccination: A Population-Based Cohort Study. *BJOG: An International Journal of Obstetrics & Gynaecology*.

[58] Goh O., Pang D., Tan J. (2023). mRNA SARS-CoV-2 Vaccination before vs during Pregnancy and Omicron Infection Among Infants. *JAMA Network Open*.

[59] Lindsay L., Calvert C., Shi T. (2023). Neonatal and Maternal Outcomes Following SARS-CoV-2 Infection and COVID-19 Vaccination: a Population-Based Matched Cohort Study. *Nature Communications*.

[60] Norman M., Magnus M. C., Söderling J. (2024). Neonatal Outcomes after COVID-19 Vaccination in Pregnancy. *JAMA*.

[61] Jorgensen S., Drover S., Fell D. B. (2023). Newborn and Early Infant Outcomes Following Maternal COVID-19 Vaccination during Pregnancy. *JAMA Pediatrics*.

[62] Zhao Y., Zhao Y., Su X. (2023). No Association of Vaccination with Inactivated COVID-19 Vaccines before Conception with Pregnancy Complications and Adverse Birth Outcomes: A Cohort Study of 5457 Chinese Pregnant Women. *Journal of Medical Virology*.

[63] Du T., Qu Q., Zhang Y., Huang Q. (2023). No Observable Influence of COVID-19 Inactivated Vaccines on Pregnancy and Birth Outcomes in the First Trimester of Gestation. *Expert Review of Vaccines*.

[64] Peretz-Machluf R., Hirsh-Yechezkel G., Zaslavsky-Paltiel I. (2022). Obstetric and Neonatal Outcomes Following COVID-19 Vaccination in Pregnancy. *Journal of Clinical Medicine*.

[65] Zhang M., Wu S., Wang D. (2024). Obstetric Outcomes of Women Vaccinated with the COVID-19 Vaccine (≥1 Dose) A Single-Center Retrospective Cohort Study of Pregnant Chinese Women. *Medicine*.

[66] Hatami D., Habibelahi A., Changizi N. (2024). Perinatal Outcomes and Sinopharm BBIBP-CorV Vaccination during Pregnancy. *BMC Pregnancy and Childbirth*.

[67] Theiler R. N., Wick M., Mehta R., Weaver A. L., Virk A., Swift M. (2021). Pregnancy and Birth Outcomes after SARS-CoV-2 Vaccination in Pregnancy. *American Journal of Obstetrics & Gynecology MFM*.

[68] Faherty E., Wilkins K. J., Jones S. (2024). Pregnancy Outcomes Among Pregnant Persons after COVID-19 Vaccination: Assessing Vaccine Safety in Retrospective Cohort Analysis of U.S. National COVID Cohort Collaborative (N3C). *Vaccines*.

[69] Nguanboonmak A., Kongsamlee R., Laohakanchanapaiboon J. (2023). Pregnancy Outcomes in COVID-19 Vaccination during Pregnancy. *Journal of the Medical Association of Thailand*.

[70] Morgan J. A., Biggio J. R., Martin J. K. (2023). Pregnancy Outcomes in Patients after Completion of the mRNA Coronavirus Disease 2019 (COVID-19) Vaccination Series Compared with Unvaccinated Patients. *Obstetrics & Gynecology*.

[71] Wainstock T., Yoles I., Sergienko R., Sheiner E. (2021). Prenatal Maternal COVID-19 Vaccination and Pregnancy Outcomes. *Vaccine*.

[72] Cai Y., Wu S., Zhang S. (2024). Prenatal Maternal Inactivated COVID-19 Vaccination: the Maternal and Neonatal Outcomes, a Retrospective Cohort Study. *Frontiers in Pharmacology*.

[73] McClymont E., Atkinson A., Albert A. (2023). Reactogenicity, Pregnancy Outcomes, and SARS-CoV-2 Infection Following COVID-19 Vaccination during Pregnancy in Canada: A National Prospective Cohort Study. *Vaccine*.

[74] Lipkind H. S., Vazquez-Benitez G., DeSilva M. (2022). Receipt of COVID-19 Vaccine during Pregnancy and Preterm or Small-For-Gestational-Age at Birth-Eight Integrated Health Care Organizations, United States, December 15, 2020-July 22, 2021. *MMWR. Morbidity and Mortality Weekly Report*.

[75] Darwin K. C., Kohn J. R., Shippey E., Uribe K. A., Gaur P., Eke A. C. (2023). Reduction in Preterm Birth Among COVID-19–Vaccinated Pregnant Individuals in the United States. *American Journal of Obstetrics & Gynecology MFM*.

[76] Hui L., Barrientos M. M., Rolnic D. L. (2023). Reductions in Stillbirths and Preterm Birth in Covid-19 Vaccinated Women: A Multi-Centre Cohort Study of Vaccine Uptake and Perinatal Outcomes. *Journal of Paediatrics and Child Health*.

[77] Hui L., Marzan M. B., Rolnik D. L. (2023). Reductions in Stillbirths and Preterm Birth in COVID-19-Vaccinated Women: a Multicenter Cohort Study of Vaccination Uptake and Perinatal Outcomes. *American Journal of Obstetrics and Gynecology*.

[78] Fell D. B., Dimanlig-Cruz S., Regan A. K. (2022). Risk of Preterm Birth, Small for Gestational Age at Birth, and Stillbirth after Covid-19 Vaccination during Pregnancy: Population Based Retrospective Cohort Study. *BMJ*.

[79] Ma Y., Shan Z., Gu Y., Huang Y. (2023). Safety and Efficacy of Inactivated COVID-19 Vaccines in Women Vaccinated during the First Trimester of Pregnancy. *International Journal of Infectious Diseases*.

[80] Lu L., Wang L., Feng T., Du X. (2023). Safety Evaluation of COVID-19 Vaccination during Early Pregnancy: A Single-Center Prospective Cohort Study of Chinese Pregnant Women. *Human Vaccines & Immunotherapeutics*.

[81] Kugelman N., Riskin A., Kedar R., Riskin‐Mashiah S. (2023). Safety of COVID-19 Vaccination in Pregnant Women: A Study of the Adverse Perinatal Outcomes. *International Journal of Gynecology & Obstetrics*.

[82] Dick A., Rosenbloom J. I., Gutman-Ido E., Lessans N., Cahen-Peretz A., Chill H. H. (2022). Safety of SARS-CoV-2 Vaccination during Pregnancy-Obstetric Outcomes from a Large Cohort Study. *BMC Pregnancy and Childbirth*.

[83] Dick A., Rosenbloom J. I., Karavani G., Gutman-Ido E., Lessans N., Chill H. H. (2022). Safety of Third SARS-CoV-2 Vaccine (Booster Dose) during Pregnancy. *American Journal of Obstetrics & Gynecology MFM*.

[84] Shanes E. D., Otero S., Mithal L. B., Mupanomunda C. A., Miller E. S., Goldstein J. A. (2021). Severe Acute Respiratory Syndrome Coronavirus 2 (SARS-CoV-2) Vaccination in Pregnancy: Measures of Immunity and Placental Histopathology. *Obstetrics & Gynecology*.

[85] Condon M., Smith N., Ayyash M., Goyert G. (2023). The Impact of COVID-19 Vaccinations on Stillbirth Rates Among Pregnant Women in the Metro-Detroit Area. *Journal of the National Medical Association*.

[86] Iannaccone A., Gellhaus A., Reisch B. (2024). The Importance of Vaccination, Variants and Time Point of SARS-CoV-2 Infection in Pregnancy for Stillbirth and Preterm Birth Risk: An Analysis of the CRONOS Register Study. *Journal of Clinical Medicine*.

[87] Citu I. M., Citu C., Gorun F. (2022). The Risk of Spontaneous Abortion Does Not Increase Following First Trimester mRNA COVID-19 Vaccination. *Journal of Clinical Medicine*.

[88] Corsi D. E., Salvatore M. A., Mandolini D. (2023). Vaccination against SARS-CoV-2 in Pregnancy during the Omicron Wave: the Prospective Cohort Study of the Italian Obstetric Surveillance System. *Clinical Microbiology and Infections*.

[89] Changizi N., Eshrati B., Salehi M. (2023). Vaccination Effects on Reducing COVID-19 Complications in Pregnancy: A Large-Scale Report from Iran. *International Journal of Gynecology & Obstetrics*.

[90] Ozer E., Cagliyan E., Cagaptay S. (2023). Vaccination Is Preventing Development of Placental Pathologies in SARS-CoV-2 Infected Pregnant Patients. *Journal of Neonatal-Perinatal Medicine*.

[91] Paixao E. S., Wong K. L. M., Alves F. J. O. (2022). CoronaVac Vaccine Is Effective in Preventing Symptomatic and Severe COVID-19 in Pregnant Women in Brazil: a Test-Negative Case-Control Study. *BMC Medicine*.

[92] Magnus M. C., Gjessing H. K., Eide H. N., Wilcox A. J., Fell D. B., Håberg S. E. (2021). Covid-19 Vaccination during Pregnancy and First-Trimester Miscarriage. *New England Journal of Medicine*.

[93] Vazquez-Benitez G., Haapala J. L., Lipkind H. S. (2023). COVID-19 Vaccine Safety Surveillance in Early Pregnancy in the United States: Design Factors Affecting the Association between Vaccine and Spontaneous Abortion. *American Journal of Epidemiology*.

[94] Danino D., Ashkenazi-Hoffnung L., Diaz A. (2023). Effectiveness of BNT162b2 Vaccination during Pregnancy in Preventing Hospitalization for Severe Acute Respiratory Syndrome Coronavirus 2 in Infants. *The Journal of Pediatrics*.

[95] Zerbo O., Ray G. T., Fireman B. (2023). Maternal SARS-CoV-2 Vaccination and Infant Protection against SARS-CoV-2 during the First Six Months of Life. *Nature Communications*.

[96] Halasa N. B., Olson S. M., Staat M. A. (2022). Maternal Vaccination and Risk of Hospitalization for Covid-19 Among Infants. *New England Journal of Medicine*.

[97] Law K. S., Hsu Y. T., Chen H. P. (2023). Preliminary Results ofCOVID ‐19 Vaccination Among Taiwanese Pregnant Women: A Single‐center, Prospective, Case–Control Study. *International Journal of Gynecology & Obstetrics*.

[98] Butt A. A., Chemaitelly H., Al Khal A. (2021). SARS-CoV-2 Vaccine Effectiveness in Preventing Confirmed Infection in Pregnant Women. *Journal of Clinical Investigation*.

[99] Kharbanda E. O., Haapala J., DeSilva M. (2021). Spontaneous Abortion Following COVID-19 Vaccination during Pregnancy. *JAMA*.

[100] Jafari M., Pormohammad A., Sheikh Neshin S. A. (2021). Clinical Characteristics and Outcomes of Pregnant Women with COVID-19 and Comparison with Control Patients: A Systematic Review and Meta-Analysis. *Reviews in Medical Virology*.

[101] Karasek D., Baer R. J., McLemore M. R. (2021). The Association of COVID-19 Infection in Pregnancy with Preterm Birth: A Retrospective Cohort Study in California. *The Lancet Regional Health-Americas*.

[102] Regan A. K., Arah O. A., Fell D. B., Sullivan S. G. (2022). SARS-CoV-2 Infection during Pregnancy and Associated Perinatal Health Outcomes: A National US Cohort Study. *The Journal of Infectious Diseases*.

[103] Lai J., Romero R., Tarca A. L. (2021). SARS-CoV-2 and the Subsequent Development of Preeclampsia and Preterm Birth: Evidence of a Dose-Response Relationship Supporting Causality. *American Journal of Obstetrics and Gynecology*.

[104] Wei S. Q., Bilodeau-Bertrand M., Liu S., Auger N. (2021). The Impact of COVID-19 on Pregnancy Outcomes: a Systematic Review and Meta-Analysis. *Canadian Medical Association Journal*.

[105] Chmielewska B., Barratt I., Townsend R. (2021). Effects of the COVID-19 Pandemic on Maternal and Perinatal Outcomes: a Systematic Review and Meta-Analysis. *Lancet Global Health*.

[106] Yao X. D., Zhu L. J., Yin J., Wen J. (2022). Impacts of COVID-19 Pandemic on Preterm Birth: a Systematic Review and Meta-Analysis. *Public Health*.

[107] Nicholas J., Roberts F., Wise A., Blair A., Mathers R., Jones R. (2023). P170 Coagulopathy and Placentitis Complicating COVID-19 Infection in the Obstetric Population. *International Journal of Obstetric Anesthesia*.

[108] Medel-Martinez A., Paules C., Peran M. (2023). Placental Infection Associated with SARS-CoV-2 Wildtype Variant and Variants of Concern. *Viruses*.

[109] Nielsen S. Y., Hvidman L. E., Aabakke A. (2023). SARS-CoV-2 Placentitis and Severe Pregnancy Outcome after Maternal Infection: A Danish Case Series. *Acta Obstetricia et Gynecologica Scandinavica*.

[110] Alcover N., Regiroli G., Benachi A., Vauloup-Fellous C., Vivanti A. J., De Luca D. (2023). Systematic Review and Synthesis of Stillbirths and Late Miscarriages Following SARS-CoV-2 Infections. *American Journal of Obstetrics and Gynecology*.

[111] Conyers S. A., Schonberg E., Powers O. (2023). Invited Speaker Talks. *American Journal of Reproductive Immunology*.

[112] Hsiao H. M., DiMaggio L. S., Perez M. A. (2023). SARS-CoV-2 Antibody Profiles in Maternal Serum and Breast Milk Following mRNA COVID-19 Vaccination: A Longitudinal Prospective Observational Cohort Study. *Vaccines*.

[113] Otero S., Miller E. S., Sunderraj A. (2023). Maternal Antibody Response and Transplacental Transfer Following Severe Acute Respiratory Syndrome Coronavirus 2 Infection or Vaccination in Pregnancy. *Clinical Infectious Diseases*.

[114] Brebant D., Couffignal C., Manchon P. (2023). Transplacental Transfer of Anti-SARS-CoV-2 Neutralizing Antibodies in Comparison to Other Pathogens Total Antibodies. *Journal of Clinical Virology*.

[115] Ware J., McElhinney K., Latham T. (2023). 8th ABM/EABM European Regional Conference May 11–13, 2023 Split, Croatia. *Breastfeeding Medicine*.

[116] Atyeo C., Shook L. L., Nziza N. (2023). COVID-19 Booster Dose Induces Robust Antibody Response in Pregnant, Lactating, and Nonpregnant Women. *American Journal of Obstetrics and Gynecology*.

[117] Kammoun M., Jarraya A., Ellouze Y. (2023). The Impact of COVID-19 Booster Vaccination in the Current Pregnancy during the Omicron Waves on Maternal and Perinatal Outcomes: a Multicentre Observational Study. *Italian Journal of Gynaecology and Obstetrics*.

[118] Barda N., Dagan N., Cohen C. (2021). Effectiveness of a Third Dose of the BNT162b2 mRNA COVID-19 Vaccine for Preventing Severe Outcomes in Israel: an Observational Study. *The Lancet*.

[119] Mbaeyi S., Oliver S. E., Collins J. P. (2021). The Advisory Committee on Immunization Practices’ Interim Recommendations for Additional Primary and Booster Doses of COVID-19 Vaccines—United States, 2021. *MMWR. Morbidity and Mortality Weekly Report*.

[120] Fernández-García S., del Campo-Albendea L., Sambamoorthi D. (2024). Effectiveness and Safety of COVID-19 Vaccines on Maternal and Perinatal Outcomes: a Systematic Review and Meta-Analysis. *BMJ Global Health*.

[121] Rahmati M., Yon D. K., Lee S. W. (2023). Effects of COVID-19 Vaccination during Pregnancy on SARS-CoV-2 Infection and Maternal and Neonatal Outcomes: A Systematic Review and Meta-Analysis. *Reviews in Medical Virology*.

[122] Beladiya J., Kumar A., Vasava Y. (2024). Safety and Efficacy of COVID-19 Vaccines: A Systematic Review and Meta-Analysis of Controlled and Randomized Clinical Trials. *Reviews in Medical Virology*.

[123] Wu S., Wang L., Dong J. (2023). The Dose- and Time-dependent Effectiveness and Safety Associated with COVID-19 Vaccination during Pregnancy: a Systematic Review and Meta-Analysis. *International Journal of Infectious Diseases*.

[124] Smith E. R., Oakley E., Grandner G. W. (2023). Clinical Risk Factors of Adverse Outcomes Among Women with COVID-19 in the Pregnancy and Postpartum Period: a Sequential, Prospective Meta-Analysis. *American Journal of Obstetrics and Gynecology*.

