# Supplementary material for: The Effectiveness and Influence of COVID-19 Vaccination on Perinatal Individuals and Their Newborns: An Updated Meta-Analysis
Source: Can J Infect Dis Med Microbiol. 2025 Jun 24;2025:6115890. doi: 10.1155/cjid/6115890 (PMC12213046; doi:10.1155/cjid/6115890)
Supplement: Supporting Information — Additional supporting information can be found online in the Supporting Information section. [file 6115890.f1.docx]

# Supplementary Material

The Effectiveness and Influence of COVID-19 Vaccination on Perinatal Women and Their Neonates: An Updated Meta-analysis

Table of contents

[Supplementary Material 1](#_Toc4821)

[Search Strategy 2](#_Toc22513)

[Embase 2](#_Toc11631)

[Cochrane 2](#_Toc19056)

[Pubmed 3](#_Toc14130)

[Scopus 4](#_Toc4366)

[Supplementary Figure 1. Maternal infection (booster vs 2 doses) 5](#_Toc8383)

[Supplementary Figure 2. Maternal infection (vaccinated during pregnancy VS vaccinated before pregnancy and unvaccinated) 6](#_Toc24783)

[Supplementary Figure 3. Maternal hospitalization (booster vs unvaccinated) 7](#_Toc15980)

[Supplementary Figure 4. Maternal hospitalization (Delta waves) 8](#_Toc19102)

[Supplementary Figure 5. Maternal hospitalization (Omicron waves) 9](#_Toc31642)

[Supplementary Figure 6. Maternal hospitalization (Booster in Delta waves) 10](#_Toc23002)

[Supplementary Figure 7. Maternal hospitalization (Booster in Omicron waves) 11](#_Toc21932)

[Supplementary Figure 8. Maternal hospitalization (2 dose) 12](#_Toc8177)

[Supplementary Figure 9. NICU (booster vs unvaccinated) 13](#_Toc17312)

[Supplementary Figure 10. Neonatal infection (2 doses) 14](#_Toc24072)

[Supplementary Figure 11. Neonatal infection (booster vs unvaccinated) 15](#_Toc6051)

[Supplementary Figure 12. Neonatal hospitalization (mRNA vaccine) 16](#_Toc17612)

[Supplementary Figure 13. Neonatal hospitalization (1st trimester) 17](#_Toc4629)

[Supplementary Figure 14. Neonatal hospitalization (2 dose) 18](#_Toc17372)

[Supplementary Figure 15. Stillbirth (booster vs unvaccinated) 19](#_Toc22998)

[Supplementary Figure 16. Preterm (booster vs unvaccinated) 20](#_Toc28010)

[Supplementary Figure 17. Preterm (<34 weeks) 21](#_Toc27606)

[Supplementary Figure 18. Intrauterine fetal death (booster vs unvaccinated) 22](#_Toc22537)

[Supplementary Figure 19. Postpartum hemorrhage (booster vs unvaccinated) 23](#_Toc25657)

[Supplementary Figure 20. Abortion (vaccineted in 1st trimester vs unvaccinated) 24](#_Toc30656)

[Supplementary Figure 21. Abortion (vaccinated during pregnancy VS vaccinated before pregnancy and unvaccinated) 25](#_Toc21906)

[Supplementary Table 1. Quality evaluation of the eligible cohort studies with Newcastle–Ottawa scale. 26](#_Toc28889)

[Supplementary Table 2. Quality evaluation of the eligible case-control studies with Newcastle–Ottawa scale. 30](#_Toc18176)

## Search Strategy

### Embase

#1"covid 19":ti,ab OR "sars cov 2":ti,ab OR "sars cov 2":ti,ab OR "Severe Acute Respiratory Syndrome Coronavirus 2":ti,ab OR "NCOV":ti,ab OR "covid 19":ti,ab OR "2019-nCoV":ti,ab OR "Coronavirus Disease 2019":ti,ab OR "SARS Coronavirus 2":ti,ab OR "2019 Novel Coronavirus":ti,ab

#2"booster":ti,ab OR vaccin*:ti,ab OR ’third dose':ti,ab

#3"miscarriage":ti,ab OR "spontaneous abortion":ti,ab OR "complication":ti,ab OR "preterm":ti,ab OR "stillbirth":ti,ab OR "neonatal death":ti,ab OR "congenital malformation":ti,ab OR "postpartum hemorrhage":ti,ab OR "antenatal bleeding":ti,ab OR "Small for gestational age":ti,ab OR "apgar":ti,ab OR "apgars":ti,ab OR "birth weight":ti,ab OR "birthweight":ti,ab OR "birthweights":ti,ab OR "Congenital anomalies":ti,ab OR "NICU admission":ti,ab OR placent*:ti,ab OR pregnan*:ti,ab OR "conception":ti,ab OR "Breastfeeding":ti,ab OR prenatal*:ti,ab OR neonatal*:ti,ab OR "maternal":ti,ab OR "maternal-fetal":ti,ab OR parturition*:ti,ab OR gestation*:ti,ab OR "Fetus":ti,ab OR "Newborn":ti,ab

#1 AND #2 AND #3

#4 'case control study'/de OR 'case report'/de OR 'case study'/de OR 'clinical article'/de OR 'clinical trial'/de OR 'clinical trial topic'/de OR 'cohort analysis'/de OR 'comparative effectiveness'/de OR 'controlled study'/de OR 'cross sectional study'/de OR 'human experiment'/de OR 'longitudinal study'/de OR 'major clinical study'/de OR 'medical record review'/de OR 'multicenter study'/de OR 'normal human'/de OR 'observational study'/de OR 'phase 1 clinical trial topic'/de OR 'phase 2 clinical trial topic'/de OR 'phase 3 clinical trial topic'/de OR 'prospective study'/de OR 'qualitative research'/de OR 'quality control'/de OR 'randomized controlled trial'/de OR 'randomized controlled trial topic'/de OR 'retrospective study'/de

### Cochrane

#1 MeSH descriptor: [COVID-19] explode all trees

#2 (SARS-CoV-2):ti,ab,kw OR (SARS CoV 2):ti,ab,kw OR (sars-cov-2):ti,ab,kw OR (Severe Acute Respiratory Syndrome Coronavirus 2):ti,ab,kw OR (NCOV):ti,ab,kw OR (2019 NCOV):ti,ab,kw OR (COVID 19):ti,ab,kw OR (2019 nCoV):ti,ab,kw OR (Coronavirus Disease 2019):ti,ab,kw OR (SARS Coronavirus 2):ti,ab,kw OR (2019 Novel Coronavirus):ti,ab,kw

#3 MeSH descriptor: [Vaccines] explode all trees

#4 (vaccin*):ti,ab,kw

#5 (booster):ab,ti,kw

#6 MeSH descriptor: [Immunization, Secondary] explode all trees

#7 (third dose):ab,ti,kw

#8 MeSH descriptor: [Pregnancy] explode all trees

#9 (pregnan*):ti,ab,kw

#10 (prenatal*):ti,ab,kw OR (neonatal*):ti,ab,kw OR (maternal ):ti,ab,kw OR (maternal-fetal):ti,ab,kw OR (gestation*):ti,ab,kw OR (parturition*):ti,ab,kw

#11 MeSH descriptor: [Abortion, Spontaneous] explode all trees

#12 miscarriage:ti,ab,kw

#13 MeSH descriptor: [Abortion, Threatened] explode all trees

#14 MeSH descriptor: [Premature Birth] explode all trees

#15 MeSH descriptor: [Infant, Extremely Premature] explode all trees

#16 MeSH descriptor: [Stillbirth] explode all trees

#17 MeSH descriptor: [Fetal Membranes, Premature Rupture] explode all trees

#18 MeSH descriptor: [Placenta] explode all trees

#19 MeSH descriptor: [Intensive Care Units, Neonatal] explode all trees

#20 (Congenital anomalies):ti,ab,kw OR (congenital malformation):ti,ab,kw 2952

#21 MeSH descriptor: [Perinatal Death] explode all trees

#22 MeSH descriptor: [Pregnancy Outcome] explode all trees

#23 (NICU):ti,ab,kw 3398

#24 MeSH descriptor: [Postpartum Hemorrhage] explode all trees

#25 MeSH descriptor: [Birth Weight] explode all trees

#26 MeSH descriptor: [Infant, Small for Gestational Age] explode all trees

#27 MeSH descriptor: [Infant, Large for Gestational Age] explode all trees

#28 MeSH descriptor: [Apgar Score] explode all trees

#29 (#1 OR #2) AND (#3 OR #4 OR #5 OR #6 OR #7) AND (#8 OR #9 OR #10 OR #11 OR #12 OR #13 OR #14 OR #15 OR #16 OR #17 OR #18 OR #19 OR #20 OR #21 OR #22 OR #23 OR #24 OR #25 OR #26 OR #27 OR #28) 267

### Pubmed

#1"covid 19"[MeSH Terms] OR "sars cov 2"[Title/Abstract] OR "sars cov 2"[Title/Abstract] OR "Severe Acute Respiratory Syndrome Coronavirus 2"[Title/Abstract] OR "NCOV"[Title/Abstract] OR "covid 19"[Title/Abstract] OR "2019-nCoV"[Title/Abstract] OR "Coronavirus Disease 2019"[Title/Abstract] OR "SARS Coronavirus 2"[Title/Abstract] OR "2019 Novel Coronavirus"[Title/Abstract]

#2"booster"[Title/Abstract] OR vaccine*[Title/Abstract] OR "Vaccines"[MeSH Terms] OR Vaccination[Title/Abstract] OR “third dose”[Title/Abstract]

#3"miscarriage"[All Fields] OR "spontaneous abortion"[All Fields] OR "Abortion, Spontaneous"[Mesh] AND "Abortion, Threatened"[Mesh] OR "complication"[All Fields] OR "preterm"[All Fields] OR "premature birth"[MeSH Terms] OR "Premature Birth"[MeSH Major Topic] AND "Premature Birth"[MeSH Terms] AND "obstetric labor, premature"[MeSH Terms] OR "infant, extremely premature"[MeSH Terms] OR "infant, premature"[MeSH Terms] OR "fetal membranes, premature rupture"[MeSH Terms] OR "Preterm Premature Rupture of the Membranes"[Supplementary Concept]

OR "stillbirth"[All Fields] OR "Stillbirth"[Mesh] AND "Pregnancy Outcome"[Mesh] OR "neonatal death"[All Fields] OR "congenital malformation"[All Fields] OR "postpartum hemorrhage"[All Fields] OR "Postpartum Hemorrhage"[Mesh] OR "antenatal bleeding"[All Fields] OR "Small for gestational age"[All Fields] OR "Infant, Small for Gestational Age"[Mesh] OR "apgar"[All Fields] OR "Apgar Score"[Mesh] OR "birth weight"[MeSH Terms] OR "birth weight"[All Fields] OR "birthweight"[All Fields] OR "birthweights"[All Fields] OR "Congenital anomalies"[All Fields] OR "NICU admission"[All Fields] OR "pregnan*"[Title/Abstract] OR "Breastfeeding"[Title/Abstract] OR "prenatal*"[Title/Abstract] OR "neonatal*"[Title/Abstract] OR "maternal"[Title/Abstract] OR "maternal-fetal"[Title/Abstract] OR "parturition*"[Title/Abstract] OR "gestation*"[Title/Abstract] OR "Fetus"[Title/Abstract] OR "Newborn"[Title/Abstract]

### Scopus

#1covid 19 OR sars cov 2 OR sars cov 2 OR Severe Acute Respiratory Syndrome Coronavirus 2 OR NCOV OR covid 19 OR 2019-nCoV OR Coronavirus Disease 2019 OR SARS Coronavirus 2 OR 2019 Novel Coronavirus

#2booster OR vaccin* OR third dose

#3miscarriage OR spontaneous abortion OR complication OR preterm OR stillbirth OR neonatal death OR congenital malformation OR postpartum hemorrhage OR antenatal bleeding OR Small for gestational age OR birth weight OR birthweight OR Congenital anomalies OR NICU admission OR placent* OR pregnan* OR conception OR Breastfeeding OR prenatal* OR neonatal* OR maternal OR maternal-fetal OR parturition* OR gestation* OR Fetus OR Newborn

## Supplementary Figure 1. Maternal infection (booster vs 2 doses)

## Supplementary Figure 2. Maternal infection (vaccinated during pregnancy VS vaccinated before pregnancy and unvaccinated)

#

## Supplementary Figure 3. Maternal hospitalization (booster vs unvaccinated)

#

## Supplementary Figure 4. Maternal hospitalization (Delta waves)

## Supplementary Figure 5. Maternal hospitalization (Omicron waves)

## Supplementary Figure 6. Maternal hospitalization (Booster in Delta waves)

## Supplementary Figure 7. Maternal hospitalization (Booster in Omicron waves)

## Supplementary Figure 8. Maternal hospitalization (2 dose)

## Supplementary Figure 9. NICU (booster vs unvaccinated)

## Supplementary Figure 10. Neonatal infection (2 doses)

## Supplementary Figure 11. Neonatal infection (booster vs unvaccinated)

## Supplementary Figure 12. Neonatal hospitalization (mRNA vaccine)

## Supplementary Figure 13. Neonatal hospitalization (1st trimester)

## Supplementary Figure 14. Neonatal hospitalization (2 dose)

## Supplementary Figure 15. Stillbirth (booster vs unvaccinated)

## Supplementary Figure 16. Preterm (booster vs unvaccinated)

## Supplementary Figure 17. Preterm (<34 weeks)

## Supplementary Figure 18. Intrauterine fetal death (booster vs unvaccinated)

## Supplementary Figure 19. Postpartum hemorrhage (booster vs unvaccinated)

## Supplementary Figure 20. Abortion (vaccineted in 1st trimester vs unvaccinated)

## Supplementary Figure 21. Abortion (vaccinated during pregnancy VS vaccinated before pregnancy and unvaccinated)

## Supplementary Table 1. Quality evaluation of the eligible cohort studies with Newcastle–Ottawa scale.

| Study | Selection | | | | Comparability | | Outcome | | |  |
| --- | --- | --- | --- | --- | --- | --- | --- | --- | --- | --- |
|  | Representative-ness | Selection of  non-exposed | Ascertainment  of exposure | Outcome not present at start | Comparability on most important factors | Comparability on other risk factors | Assessment of outcome | Long enough follow-up median≥1 year | Adequacy  completeness of follow-up |  |
| Minghui et al. | 6- | * | * | - | - | * | * | * | * |  |
| L. Lindsay et al. | 9* | * | * | * | * | * | * | * | * |  |
| Raanan Meyer et al. | 9* | * | * | * | * | * | * | * | * |  |
| A. Nguanboonmak et al. | 9* | * | * | * | * | * | * | * | * |  |
| E. Ozer et al. | 7- | * | * | * | - | * | * | * | * |  |
| M. Rottenstreich(2023) et al. | 9* | * | * | * | * | * | * | * | * |  |
| H. Süt et al. | 7- | * | * | * | - | * | * | * | * |  |
| Z. Teng et al. | 7- | * | * | * | * | * | * | * | - |  |
| G. S. Tripathy et al. | 8- | * | * | * | * | * | * | * | * |  |
| M. Zare Sakhvidi et al. | 8* | * | * | * | - | * | * | * | * |  |
| O. Zerbo(2023)et al. | 9* | * | * | * | * | * | * | * | * |  |
| M.Lipschuetz et al. | 9* | * | * | * | * | * | * | * | * |  |
| O.Zerbo(2023)(2)et al. | 9* | * | * | * | * | * | * | * | * |  |
| Y. Zhao et al. | 9* | * | * | * | * | * | * | * | * |  |
| Y.Ma et al. | 7* | * | - | * | * | * | * | * | - |  |
| J.A. Morgan(2023) et al. | 8* | * | * | * | - | * | * | * | * |  |
| L.Lu et al. | 8* | * | * | * | - | * | * | * | * |  |
| L.Hui(2023)(1)et al. | 8* | * | * | * | - | * | * | * | * |  |
| E.Ibroci et al. | 8* | * | * | * | - | * | * | * | - |  |
| H. Kim(2023) et al. | 6- | * | * | * | - | - | * | * | * |  |
| N.Kugelman et al | 8* | * | * | * | - | * | * | * | * |  |
| A. Jarraya(2023) et al. | 8* | * | * | * | - | * | * | * | * |  |
| N. Changizi et al. | 7* | * | * | * | - | * | * | * | - |  |
| L. Ghesquiere et al. | 9* | * | * | * | * | * | * | * | * |  |
| E. McClymont et al. | 9* | * | * | * | * | * | * | * | * |  |
| J. J. Yland et al. | 7* | * | - | * | * | * | - | * | * |  |
| S. N. Piekos et al. | 9* | * | * | * | * | * | * | * | * |  |
| Noa Dagan et al.(30) | 9* | * | * | * | * | * | * | * | * |  |
| M Rottenstreich(2022) et al. | 9* | * | * | * | * | * | * | * | * |  |
| R. N. Theiler et al. | 7* | * | * | * | - | - | * | * | * |  |
| T.Wainstock et al. | 9* | * | * | * | * | * | * | * | * |  |
| D.B. Fell(2022)(1)et al. | 8* | * | * | * | - | * | * | * | * |  |
| M.C. Magnus et al. | 9* | * | * | * | * | * | * | * | * |  |
| H.S. Lipkind et al. | 8* | * | * | * | - | * | * | * | * |  |
| Orlanda Goh et al. | 8* | * | * | * | - | * | * | * | * |  |
| H.Blakeway et al. | 8* | * | * | * | - | * | * | * | * |  |
| Z.Chen et al. | 9* | * | * | * | * | * | * | * | * |  |
| J.A. Morgan(2022) et al. | 8* | * | * | * | - | * | * | * | * |  |
| T.Du et al. | 9* | * | * | * | * | * | * | * | * |  |
| C.Yang et al. | 7- | * | * | * | - | * | * | * | * |  |
| Aharon Dick et al.(1) | 9* | * | * | * | * | * | * | * | * |  |
| Aharon Dick et al.(2) | 8* | * | * | * | * | * | * | * | - |  |
| Inbal Goldshtein et al. | 7* | * | * | * | * | * | * | - | - |  |
| Inbal Goldshtein et al.(2) | 9* | * | * | * | * | * | * | * | * |  |
| Ravit Peretz-Machluf et al. | 9* | * | * | * | * | * | * | * | * |  |
| Boelig et al. | 7- | * | - | * | * | * | * | * | * |  |
| Shanes et al. | 7- | * | - | * | * | * | * | * | * |  |
| Ellen Øen Carlsen et al. | 9* | * | * | * | * | * | * | * | * |  |
| D.B Fell(2022)(2) et al. | 8* | * | * | * | - | * | * | * | * |  |
| L. Hui(2023)(2) et al. | 8* | * | * | * | - | * | * | * | * |  |
| Ioana Mihaela Citu et al. | 8* | * | * | * | - | * | * | * | * |  |
| Clara Calvert et al. | 9* | * | * | * | * | * | * | * | * |  |
| Eunseon Gwak et al. | 9* | * | * | * | * | * | * | * | * |  |
| Sarah C. J. Jorgensen et al. | 8* | * | * | * | - | * | * | * | * |  |
| J. Guedalia | 9* | * | * | * | * | * | * | * | * |  |
| Condon M. et al. | 8* | * | * | * | - | * | * | * | * |  |
| Corsi Decenti E. et al. | 8* | * | * | * | - | * | * | * | * |  |
| Darwin K,C. et al. | 8* | * | * | * | - | * | * | * | * |  |
| Du T. et al. | 8* | * | * | * | * | * | * | * | - |  |
| Barros, F. C. et al. | 8* | * | * | * | - | * | * | * | * |  |
| Cai, Y. et al. | 9* | * | * | * | * | * | * | * | * |  |
| De Virgilio Suglia, C. et al. | 8* | * | * | * | - | * | * | * | * |  |
| Faherty, E. A. G. et al. | 8* | * | * | * | - | * | * | * | * |  |
| Hatami, D. et al. | 8* | * | * | * | - | * | * | * | * |  |
| Iannaccone, A. et al. | 7* | * | * | * | - | - | * | * | * |  |
| Mensah et al. | 9* | * | * | * | * | * | * | * | * |  |
| Norman et al. | 9* | * | * | * | * | * | * | * | * |  |
| Santos J et al. | 8* | * | * | * | * | * | * | * | - |  |
| Arun K. Suseeladevi et al. | 9* | * | * | * | * | * | * | * | * |  |
| Velez et al. | 9* | * | * | * | * | * | * | * | * |  |
| Yeniocak AS et al. | 8* | * | * | * | - | * | * | * | * |  |
| G. Zels et al. | 7* | * | * | * | - | * | * | - | * |  |
| Zhang M et al. | 7* | * | * | * | - | * | * | - | * |  |
| A.P. Mahyuddin et al. | 8* | * | * | * | - | * | * | * | * |  |
| *indicates criterion met; - indicates significant of criterion not met. | | | | | | | | | | |

## Supplementary Table 2. Quality evaluation of the eligible case-control studies with Newcastle–Ottawa scale.

| Study | Selection | | | | Comparability | | | Outcome | | |
| --- | --- | --- | --- | --- | --- | --- | --- | --- | --- | --- |
|  | Representativeness of the case | Adequate definition of the case | Selection of  control | Definition of Controls | Comparability on most important factors | Comparability on other risk factors | Assessment of outcome | | Ascertainment of exposure | Non-Response rate |
| R. M. Simeone et al. | 8* | * | * | * | - | * | * | | * | * |
| O. Zerbo et al. | 9* | * | * | * | * | * | * | | * | * |
| Adeel A. Butt et al. | 9* | * | * | * | * | * | * | | * | * |
| Enny S. Paixao et al. | 9* | * | * | * | * | * | * | | * | * |
| Danino D et al. | 8* | * | * | * | - | * | * | | * | * |
| Halasa et a | 8* | * | * | * | - | * | * | | * | * |
| G. Vazquez-Benitez et al. | 8* | * | * | * | - | * | * | | * | * |
| Kim Seng Law et al | 6- | * | * | * | - | * | * | | * | - |
| 1. C. Magnus(2021) et al. | 8* | * | * | * | - | * | * | | * | * |
| E. O. Kharbanda et al. | 8* | * | * | * | - | * | * | | * | * |
| Gabriela et al. | 9* | * | * | * | * | * | * | | * | * |
| Denoble, A. E. et al. | 8* | * | * | * | - | * | * | | * | * |
| Mensah et al. | 9* | * | * | * | * | * | * | | * | * |
| Norman et al. | 9* | * | * | * | * | * | * | | * | * |
| *indicates criterion met; - indicates significant of criterion not met. | | | | | | | | | | |
